# Description of *Longidorus cholevae* sp. n. (Nematoda, Dorylaimida) from a riparian habitat in the Rila Mountains, Bulgaria

**DOI:** 10.3897/zookeys.330.5750

**Published:** 2013-09-09

**Authors:** Vlada K. Peneva, Stela S. Lazarova, Francesca De Luca, Derek J. F. Brown

**Affiliations:** 1 Institute of Biodiversity and Ecosystem Research, Bulgarian, Academy of Sciences, 2 Garagrin Street, 1113 Sofia, Bulgaria; 2 Plant Protection Institute, National Research Council, Via Amendola 122/D, 70126, Bari, Italy

**Keywords:** D2D3, ITS, Longidoridae, morphology, phylogeny

## Abstract

A description is provided of *Longidorus cholevae* **sp. n.**, a bisexual species associated with wild cherry (*Prunus avium* L.) from the Rila Mountains, Bulgaria. The position of *L. cholevae* sp. n. among other species of the genus was elucidated by using morphological and molecular data. Phylogenetic analyses were performed of D2-D3 expansion domains of the 28S rRNA and the partial ITS1 containing regions by Neighbor-Joining, Maximum Likelihood and Bayesian Inference methods. The species is characterised by a female body length of 6.1–8.1 mm; long odontostyle (106–129 μm); lip region wide (21.5–24 μm) rounded and continuous with the body profile; amphidial pouches short and wide, funnel-shaped; a posteriorly situated guide ring (30–37 μm); normal arrangement of pharyngeal glands, and short bluntly rounded to hemispherical tail. Four juvenile stages indentified, first stage with elongate conoid tail. Males with 2–4 adanal pairs and a row of 11–13 single ventromedian supplements, spicules 96–120 μm long. Based both on morphological and molecular data the new species appearred to be the most similar witha group of species distributed in Europe sharing common charcters such as amphidial fovea, lip region and tail shapes, and having similar odontostyle and body length: *L. poessneckensis*, *L. caespiticola*, *L. macrososma*, *L. helveticus*, *L. carniolensis* and *L. pius*. An updated list of *Longidorus* species and a partial polytomous keys to the *Longidorus* species with long odontostyle (code A45) and short tail (code H1) are provided.

## Introduction

Chen et al. (1997) further developed a polytomous key for identification of 103 species known at that time of the genus *Longidorus* Micoletzky, 1925. Subsequently, Loof and Chen (1999) provided codes for another 13 species, two of which were cosidered as junior sysnonyms. Recently, another four species, originally described either as *Paralongidorus* (e.g *Prunus monegrensis* and *Prunus milanis*) or *Longidoroides* (*Longidorus spiralis* and *Longidorus boshi*) were transferred to *Longidorus* (Roca 2006, Decraemer and Coomans 2007). Andrássy (2009) provided a list of the species belonging to the genus, noting that 69 species were registered to occur in Europe. To this list eight new species were added which originated from different parts of the world: Ukraine (*Longidorus holovachovi* Peneva, Sususlovsky & Lazarova, 2009), Slovenia (*Longidorus carniolensis* Širca, Urek, Lazarova, Elshishka & Peneva, 2011), Iran (*Longidorus kheirii* Pedram, Niknam, Robbins, Ye & Karegar, 2008 and *Longidorus tabrizicus* Niknam, Pedram, Ghahremani Nejad, Ye, Robbins & Tanha Maafi, 2010), Philippines (*Longidorus mindanaoensis* Coomans, Tandingan De Ley, Angsinco Jimenez & De Ley, 2012 and Spain (*Longidorus baeticus* Gutiérrez-Gutiérrez, Cantalapiedra-Navarrete, Monte-Borrego, Palomares-Rius & Castillo, 2013, *Longidorus oleae* Gutiérrez-Gutiérrez, Cantalapiedra-Navarrete, Monte-Borrego, Palomares-Rius & Castillo, 2013 and *Longidorus andalusicus* Gutiérrez-Gutiérrez, Cantalapiedra-Navarrete, Monte-Borrego, Palomares-Rius & Castillo, 2013). Currently, there are 158 *Longidorus* species and an updated list of the species belonging to this important plant parasitic genus is presented as Appendix 1.

Molecular approaches and phylogenetic studies provide additional tools to the routine identification of plant parasitic nematodes. Further, the ribosomal DNA sequences represent a useful diagnostic aproach in the characterisation and phylogenetic reconstruction within Longidoridae, above all, where morphological characters may led to ambiguous identification (De Luca et al. 2004, 2009, Neilson et al. 2004, He at al. 2005, Palomares-Rius et al. 2008, 2010).

During a study of the longidorid fauna of natural habitats in Bulgaria (2005-2009) several populations of the genus *Longidorus* were recovered from various locations in the Rila Mountains, one of which represented an undescribed species.

The aim of the present study was to characterise morphologically and molecularly this new species and to infer its phylogenetic relationships with other species of the genus *Longidorus* by using the D2-D3 expansion domains of the 28S rDNA and the ITS containing region.

## Materials and methods

Nematodes were isolated from soil samples by a decanting and sieving technique. *Longidorus* specimens recovered were heat killed at 55°C for two minutes, fixed in a 4% formalin/1% glycerol mixture, processed to anhydrous glycerol (Seinhorst 1959), and mounted on glass microscope slides. Drawings were prepared using an Olympus BX51 compound microscope with differential interference contrast (DIC). Photographs were taken using an Axio Imager. M2-Carl Zeiss compound microscope equipped with a digital camera (ProgRes C7) and specialised software (CapturePro Software 2.8). Measurements were made using an Olympus BX41 light microscope, a digitising tablet (CalComp Drawing Board III, GTCO CalCom Peripherals, Scottsdale, AZ, USA), and computer Digitrak 1.0f programme, (Philip Smith, Scottish Crop Research Institute, Dundee, UK).

A partial polytomous keys was prepared for the identification of *Longidorus* species with long odontostyle (A45) and short tail (H1). This key, based on that by Chen et al. (1997), but incorporating newly described species after 1997 and the addition of some new characters: J – number of juvenile stages – J1 – 4 stages; J2 – 3 stages; K – shape of tail in J1 – using the same codes as for female tail and introducing K7 – tail digitate or with mucro.

### DNA extraction and amplification

Specimens for molecular analysis were kept in DESS solution (Yoder et al. 2006). Genomic DNA was extracted from fifteen individual nematodes as described by De Luca et al. (2004). The crude DNA isolated from each individual nematode was directly amplified. The partial 18S-ITS1-5.8S-ITS2 regions were amplified using the forward primer 18S-Ext (5’-TGATTACGTCCCTGCCTTT-3’) and the reverse primer 26S-Ext (5’-TTTCACTCGCCGTTACTAAGG-3’) (Vrain et al. 1992) and the D2-D3 expansion segments of 28S rDNA was amplified using the D2A (5’-ACAAGTACCGTGAGGGAAAGTTG-3’) and D3B (5’-TCGGAAGGAACCAGCTACTA-3’) primers (Castillo et al. 2003). PCR cycling conditions used for amplification were: an initial denaturation at 94°C for 5 min, followed by 35 cycles of denaturation at 94°C for 50s, annealing at 55°C for 50s and extension at 72°C for 1 min and a final step at 72°C for 7 min. The size of amplification products was determined by comparison with the molecular weight marker ladder 100 (Fermentas, St. Leon-Rot, Germany) following electrophoresis of 10 ml on a 1% agarose gel.

### Sequencing and phylogenetic analysis

PCR products of the ITS region from two individual nematodes were purified for cloning and sequencing using the protocol provided by the manufacturer (High Pure PCR elution kit, Roche, Germany). Purified ITS fragments were cloned in TA cloning vector (Invitrogen) and several clones were sequenced using an ABI Prism 377 sequencer (PE Applied Biosystem, Foster City, CA). Similarly, the D2-D3 regions of rDNA from two individual nematodes were purified and used for direct sequencing. The sequences of the new species have been deposited in GenBank with the accession numbers: FR775757 – FR775760 for the ITS clones; and FR775761, FR775762 for the D2-D3 regions. Additionally, another four sequences (ITS and D2-D3) belonging to a population identified as *Longidorus* cf. *caespiticola* Hooper, 1961 were produced and deposited using the same methodology (see Table 1 for accession numbers and locality). The morphometrics of this population and detailed discussion will be presented in another publication.

**Table 1.** Species of fam. Longidoridae used in phylogenetic reconstructions.

**Nematode species**	**Locality**	**Accession number**	**Reference**
*Longidorus caespiticola* Hooper, 1961	Brdo, Slovenia	HM447030	Širca and Urek 2009
*Longidorus caespiticola*	Brussegem, Belgium	AF480079	Rubtsova et al. 2001
*Longidorus caespiticola*	Gandesbergen, Germany	AF480080	Rubtsova et al. 2001
*Longidorus caespiticola*	Viermaal, Belgium	AF480081	Rubtsova et al. 2001
*Longidorus caespiticola*	Scotland, UK	AY601567	He et al. 2005
*Longidorus* cf. *caespiticola*	Sokolovo, Bulgaria	HG329719–HG329721	Present study
*Longidorus carniolensis*	Krmačina, Slovenia	JN631811	Širca et al. 2011
*Longidorus carniolensis*	Drašiči, Slovenia	JN631812	Širca et al. 2011
*Longidorus cholevae* sp. n.	Bulgaria	FR775757–FR775762	Present study
*Longidorus elongatus* (de Man, 1876) Thorne & Swanger, 1936	Scotland, UK	AF511417	Ye et al. 2004
*Longidorus helveticus* Lamberi, Kunz, Grunder, Molinari, De Luca, Agostinelli & Radicci, 2001	Trška gora, Slovenia	HM447031	Širca and Urek 2009
*Longidorus helveticus*	Stari Ledinci, Serbia	EF538753, JN627412	Kumari et al. 2009<br/> Kumari and Subbotin 2012
*Longidorus helveticus*	Camenzuid, Switzerland	AY601566	He et al. 2005
*Longidorus helveticus*	Chodovlice, Czech Republic	JN627410, JN627414	Kumari and Subbotin 2012
*Longidorus helveticus*	Silničná, Czech Republic	JN627411, JN627415	Kumari and Subbotin 2012
*Longidorus helveticus*	Switzerland	AJ549985	De Luca et al. 2004
*Longidorus macrosoma* Hooper, 1961	Liége, Belgium	AF480082	Rubtsova et al. 2001
*Longidorus macrosoma*	Austria	EF538752	Kumari et al. 2009
*Longidorus macrosoma*	unknown	AY580055	unpublished
*Longidorus macrosoma*	Switzerland	AY601565	He et al. 2005
*Longidorus macrosoma*	Switzerland	AJ549978, AJ549979	De Luca et al. 2004
*Longidorus macrosoma*	unknown	AY430184	unpublished
*Longidorus pius* Barsi & Lamberti, 2000	Republic of Macedonia	AM743178–AM743184	Barsi and De Luca 2008
*Longidorus poessneckensis* Altherr, 1974	Czech Republic	EF538750	Kumari et al. 2009
*Longidorus poessneckensis*	Slovakia	EF538751	Kumari et al. 2009
*Longidorus raskii* Lamberti & Agostinelli, 1993	Switzerland	AJ549983, AJ549984	De Luca et al. 2004
*Longidorus diadecturus* Eveleigh & Allen, 1982	Elkins, White river, USA	AY601584	He et al. 2005
*Xiphinema diversicaudatum* (Micoletzky, 1927) Thorne, 1939	Slovakia	EF538755	Kumari et al. 2009
*Xiphinema index* Thorne & Allen, 1950	Argentina	AY601628	He et al. 2005
*Xiphinema insigne* Loos, 1949	Taiwan	AY563427	Chen et al. 2004

Further, a BLAST (Basic Local Alignment Search Tool) search at NCBI (National Center for Biotechnology Information) was performed using the obtainedITS and D2-D3 sequences as queries to confirm their nematode origins and to identify the most closely related nematode sequences. Different *Longidorus* species were used in the phylogenetic analyses of ITS1-5.8S-ITS2 and D2-D3 regions due to sequence availability in the GenBank database (Table 1). The multiple sequence alignments (MSA) of both datasets were performed using MAFFT algorithm (Katoh et al. 2002) with GUIDANCE Web-based program available at http://guidance.tau.ac.il/ (Penn et al. 2010a). The MSA reliability evaluation was based on GUIDANCE alignment, sequence and columns scores (Penn et al. 2010b). Unreliable columns below 0.93 confidence score were removed from the D2-D3 MSA alignment. Subsequently, the MSAs were manually optimised and trimmed using MEGA 5 (Tamura et al. 2011). *Xiphinema diversicaudatum* (Micoletzky, 1927) Thorne, 1939, *Xiphinema index* Thorne & Allen, 1950 and *Xiphinema insigne* Loos, 1949 were used as out group taxa for both D2-D3 and ITS sequence datasets, respectively.

Base compositional differences were evaluated using the c^2^-test. Sequence divergences (uncorrected *p* distance) were calculated using MEGA 5.0 (Tamura et al. 2011). The phylogenetic reconstructions of both D2-D3 and partial 18S-ITS1 rDNA datasets were performed using neighbor joining (NJ) and maximum likelihood (ML) algorithms as implemented in MEGA 5.0 (Tamura et al. 2011) as well as the Bayesian inference (BI) using MrBayes v. 3.2.1 (Huelsenbeck and Ronquist 2001, Ronquist and Huelsenbeck 2003, Ronquist et al. 2012). The *NJ phylogenetic inferences* were performed under the following settings: Maximum Composite Likelihood method for computing evolutionary distances; Gamma distributed rates among sites, estimated values set up to 0.3395 (D2-D3) and 0.1127 (18S-ITS1); 2000 bootstrap replications. A total of 640 and 290 positions in the final datasets were used for both analyses, respectively. The most appropriate substitution models were determined using the FindModel web tool (Tao et al. 2005, Posada and Crandall 1998), by comparing the Akaike information criterion (AIC, Akaike 1973) and Maximum Likelihood value (*ln* L) scores of the 28 possible models. *ML analyses* settings as applied in MEGA 5were General Time Reversible model (GTR), Gamma distribution (G); number of discrete Gamma rates equal to 4; 1000 bootstrap replications for D2-D3 rDNA and Kimura 2 parameter-model (+G, 4 rates and 1000 bootstrap replications) for 18S-ITS1 region. Bayesian MCMC tree searches were conducted using MrBayes 3.2.1. For each analysis, two independent runs were conducted with 4 chains each and default heating parameters (1 cold, 3 heated, *temp* = 0.2). Each analysis was run for 10,000,000 generations with a sample frequency of 1000 generations. The first 25% of the chains discarded as burning and the remaining 75% trees kept to summarise the tree topology, branch lengths, and posterior probabilities (PP) of branch support. The evolutionary models for nucleotide substitutions were set up as for ML analyses. Convergence diagnostic calculated every 1000 generations with predefined stopvalue equal to 0.01. A single strict consensus tree was visualised using FigTree v1.4.0 graphical viewer (http://tree.bio.ed.ac.uk/software/figtree/). Posterior probabilities values of ³0.8 (BI) and bootstrap values of ³70 (NJ and ML) were considered as credible support values for nodes.

## Taxonomy

### Longidorus cholevae sp. n.

http://zoobank.org/882B3067-D244-4B8F-9312-B6E0F55B6C90

http://species-id.net/wiki/Longidorus_cholevae

Figures 1 –9

#### Measurements.

See Table 2

**Table 2.** Measurements of females, males and juvenile stages of *Longidorus cholevae* sp. n. from Bachevo village. All measurements are given in μm (mean ± standard deviation, with range in parentheses).<br/>

–	**Holo-**	**Females**	**Males**	**J1**	**J2**	**J3**	**J4**
**n**	**type**	**11**	**11**	**9**	**8**	**9**	**11**
L	7199	6788 ± 573<br/> (6127–8083)	6390 ± 594<br/> (5415–7111)	1209 ± 63<br/> (1135–1289)	1874 ± 236<br/> (1554–2251)	3048 ± 406<br/> (2336–3447)	4798 ± 442<br/> (4148–5666)
a	83.3	72.1 ± 7.4<br/> (61.1–83.3)	70.2 ± 6.2<br/> (63.9–82.0)	47.0 ± 1.9<br/> (43.8–50.3)	51.1 ± 2.4<br/> (49.0–55.3)	56.5 ± 3.8<br/> (50.2–61.3)	63.8 ± 5.9<br/> (54.8–76.6)
b	13.1	14.3 ± 1.5<br/> (12.3–17.9)	12.7 ± 1.2<br/> (10.7–14.7)	4.5 ± 0.4<br/> (3.9–5.1)	5.8 ± 0.9<br/> (4.5–7.2)	7.9 ± 0.9<br/> (7.2–9.9)	10.9 ± 1.5<br/> (9.2–14.1)
c	202.4	199.7 ± 15.4<br/> (171.2–220.4)	199.6 ± 18.3<br/> (171.1–227.8)	29.6 ± 3.8<br/> (26.1–36.6)	48.2 ± 3.6<br/> (43.2–53.9)	78.3 ± 7.2<br/> (66.1–91.3)	136.5 ± 19.9<br/> (115.5–181.1)
c’	0.6	0.6 ± 0.06<br/> (0.5–0.7)	0.6 ± 0.06<br/> (0.6–0.8)	2.1 ± 0.19<br/> (1.8–2.4)	1.4 ± 0.1<br/> (1.2–1.5)	0.9 ± 0.07<br/> (0.8–1.0)	0.7 ± 0.06<br/> (0.6–0.8)
V (%)	52.5	50.5 ± 2.2<br/> (46.7–53.4)	-	-	-	-	-
G_1_ (%)	13.0	14.0 ± 2.8<br/> (8.6–17.7)	-	-	-	-	-
G_2_ (%)	11.5	14.2 ± 1.6<br/> (11.6–17.1)	-	-	-	-	-
d	1.3	1.3 ± 0.04<br/> (1.2–1.4)	1.3 ± 0.04<br/> (1.3–1.4)	1.7 ± 0.08<br/> (1.6–1.8)	1.6 ± 0.09<br/> (1.5–1.7)	1.6 ± 0.11<br/> (1.4–1.7)	1.5 ± 0.08<br/> (1.3–1.5)
d’	1.5	1.5 ± 0.06<br/> (1.4–1.6)	1.5 ± 0.03<br/> (1.4–1.6)	1.6 ± 0.07<br/> (1.5–1.7)	1.7 ± 0.1<br/> (1.6–1.8)	1.7 ± 0.1<br/> (1.5–1.8)	1.7 ± 0.08<br/> (1.6–1.9)
Odontostyle	121	120.1 ± 7.2<br/> (106–129)	121.2 ± 5.1<br/> (115–131)	61.1 ± 3.5<br/> (56–66)	65.9 ± 2.8<br/> (62–71)	84.7 ± 3.3<br/> (79–90)	99.1 ± 5.3<br/> (88–105)
Replacement odontostyle	-	-	-	65.0 ± 1.8<br/> (61–67)	78.1 ± 4.3<br/> (74–86.5)	101.4 ± 4.5<br/> (96–109)	117.5 ± 7.9<br/> (105.5–131)
Developing<br/> gonads	-	-	-	19.9 ± 3.2<br/> (16–25)	28.3 ± 3.2<br/> (24–34)	53.1 ± 8.7<br/> (41–65)	135.5 ± 11.4<br/> (114–147)
Odontophore	88	76.3 ± 3.3<br/> (74-81)	73.7 ± 5.0<br/> (69.5-81)	41.7 ± 5.4<br/> (36-48)	48.6 ± 3.3<br/> (42-52)	62.6 ± 2.5<br/> (60-66)	71.1 ± 3.6<br/> (67-79)
Pharynx	550	481.9 ± 47.8<br/> (439–577)	507.0 ± 45.6<br/> (421–584)	273.6 ± 19.3<br/> (250–302)	318.4 ± 23.6<br/> (277–349)	398.1 ± 47.7<br/> (311.5–450.5)	445.7 ± 38.9<br/> (362–491)
Anterior to guiding ring	36	32.6 ± 2.21<br/> (30–37)	33.5 ± 1.1<br/> (32–36)	16.2 ± 0.7<br/> (15–18)	19.3 ± 0.74<br/> (18–20)	24.2 ± 1.6<br/> (22.5–27)	28.4 ± 1.4<br/> (25.5–31)
Bulb length	139	128 ± 12.5<br/> (114.5–146.5)	124 ± 7.2<br/> (115–137)	60.7 ±5.1<br/> (53–66)	72.5 ± 8.9<br/> (65–90)	100.7 ±5.6<br/> (92–108)	116.8 ± 9.4<br/> (105–128)
Bulb width	34	34.1 ± 2.9<br/> (30–38)	33.0 ± 3.3<br/> (28–38)	15 ± 1.2<br/> (14–17)	20.2 ± 2.0<br/> (18–23)	26.3 ± 1.2<br/> (24–27)	29.8 ± 2.5<br/> (26–34)
Tail	35.5	34.1 ± 2.9<br/> (28.5–38)	32.2 ± 3.3<br/> (29–39)	41.3 ± 4.8<br/> (35–48.5)	38.8 ± 2.8<br/> (34–43)	38.9 ± 3.3<br/> (34–44.5)	35.4 ± 2.6<br/> (31–38)
Length of hyaline part	19	18.1 ± 1.10<br/> (17–20)	14.5 ± 2.2<br/> (12–18)	10.9 ± 1.8<br/> (9–14)	11.8 ± 2.09<br/> (9–15)	13.9 ± 1.6<br/> (12–17)	14.5 ± 1.4<br/> (12–16.5)
Body diameter at:<br/> - lip region	<br/> 22.5	22.8 ± 0.8<br/> (21.5–24)	23.0 ± 0.7<br/> (22–24)	9.5 ± 0.30<br/> (9–10)	12.0 ± 0.6<br/> (11–13)	15.5 ± 1.2<br/> (14–17)	19.5 ± 1.0<br/> (18–21)
- guiding ring	39	37.5 ± 2.5<br/> (35–43)	37.7 ± 1.7<br/> (34–40)	15.1 ± 0.32<br/> (14–15.5)	20.1 ± 1.09<br/> (18–22)	26.2 ± 1.8<br/> (23.5–28.3)	33.2 ± 1.7<br/> (30 - 35.5)
- base of pharynx	74.5	75.9 ± 8.4<br/> (69–100)	77.5 ± 8.8<br/> (66–90.5)	26.3 ± 2.0<br/> (24–30)	35.2 ± 2.8<br/> (31–38)	49.0 ± 4.60<br/> (41–53)	63.4 ± 5.2<br/> (57–77)
- mid-body/at vulva	86	93.7 ± 9.1<br/> (83–106)	91.7 ± 11.5<br/> (73–111)	25.9 ± 2.1<br/> (23–29.5)	36.6 ± 3.8<br/> (31–41)	54.1 ± 7.2<br/> (43.5–68)	75.6 ± 8.5<br/> (66–94)
- anus	57	54.8 ± 4.4<br/> (48–66)	52.4 ± 4.0<br/> (46–58)	19.5 ± 1.2<br/> (18–22)	28.7 ± 2.3<br/> (25–31)	42.8 ± 4.3<br/> (36–48)	52.2 ± 2.5<br/> (47–56)
- hyaline part	47	42.9 ± 3.8<br/> (37–48)	36.0 ± 4.0<br/> (27–42)	10.9 ± 1.77<br/> (9–14)	17.9 ± 2.4<br/> (14.5–21)	29.0 ± 3.4<br/> (25–37)	37.2 ± 3.0<br/> (33–42)
Spicules	-		105.9 ± 6.9<br/> (96–120)				

#### Description.

*Female*. Body plump, assuming a C to open spiral shape. Lip region continuous, anteriorly rounded. Labial papillae prominent. Cuticle 8–10 μm thick at poslabial region, 5–7 μm along the body and 12–14 μm on tail posterior to anus. Guide ring 6–7 μm wide. One lateral pore anterior to guide ring, 2–4 along odontostyle, 1–2 along odontophore, 4–5 in narrow part of the oesophagus and 3–4 in bulb region as well as 3–5 dorsal and 7–10 ventral; numerous lateral body pores observed. Amphidial fovea pouch like, short, almost as wide as long, funnel shape with code E5 according to Chen et al. (1997) and type 4 according to Decraemer and Coomans (2007), amphidial aperture assumed to be a minute pore, hardly visible under light microscope; fusus (sensillium pouch) at 51.6±2.7 (49.5–56) μm, n=7 from anterior end. Odontostyle slender, 2 μm wide at base. Pharyngo-intestinal valve, variable in shape (broadly rounded to heart-shape) and size, slightly wider than long: 19±1.4 (17–20) × 15.4±3.1 (12–19) μm, n=5. Normal arrangement of pharyngeal glands: nuclei of the dorsal and subventral glands situated at 23.6–32.1 % (n=3) and 50.7–58.9 % (n=8) of the distance from anterior end of the bulb. Dorsal gland nuclei 2 μm diam., subventral gland nuclei 3–4 μm diam. Nerve ring surrounding odontophore base, at 222.9±11.3 (203–242.5) μm from anterior end, a second nerve ring situated at a short distance behind the first one. Lateral chord 25–29 μm wide. Vagina extending to *ca.* half corresponding body width. *Pars distalis vaginae* 23–27 μm long; *pars proximalis vaginae* 28–35 μm long, thick walled. Uteri very long, anterior uterus 481.0±105.1 (372.5–662.5), posterior uterus 473.2±114.2 (357.5–660) μm long, respectively; well developed sphincter between uterus and *pars dilatata oviductus*, *pars dilatata* and uteri usually containing numerous sperm cells. Prerectum 426.9±79.7 (310–595) μm long, rectum 45.5±1.6 (43–48) μm or about 0.7–0.8 of body diameter at anus. Tail bluntly conoid, rounded to hemispherical. Two pairs of lateral pores.

*Male*. Habitus as in females, posterior part more strongly coiled ventrad. Shape of lip region similar to that in females. Cuticle 5–8 μm thick at poslabial region, 7–9 at guiding ring level, 4–6 μm along the body and 9–13 μm on tail posterior to cloaca. One lateral pore anterior to guide ring, 2–3 along odontostyle, 1–2 along odontophore, 3–5 in narrow part of the oesophagus and 3–4 in pharyngeal bulb region as 4 dorsal and 7–10 ventral; numerous lateral body pores present. Fusus at 52.3±3.7 (47–57) μm, n=7 from anterior end. Nerve ring surrounding odontophore base, at 231.8±12.2 (217.5–259.5) μm from anterior end, a second nerve ring situated at a short distance behind the first one. Pharyngo-intestinal valve, variable in shape (broadly rounded to heart-shape) and size, almost as long as wide: 16.6±3.2 (13–23) × 18±3.1 (13–22) μm, n=6. Lateral chord 20–25 μm wide. Supplements 3–4 adanal pairs followed by 10–14 arranged irregularly in a single row. Spicules massive, slightly curved ventrally, lateral guiding piece 27–28 μm long. Spermatozoids round small (4–6 μm diam.). Tail short, bluntly conoid, dorsally convex, ventrally slightly concave, three pairs of lateral pores.

*Juveniles*. Morphometrics obtained from juvenile specimens, and of the relationship between the lengths of their functional and replacement odontostyles and body lengths, confirmed the presence of four juvenile stages (Figure 9). Habitus in the shape of more or less open C, tail of the first stage juvenile conoid elongated whereas in the subsequent developmental stages the tail is conoid (second stage) to bluntly conoid (third and fourth stage).

**Figure 1.**
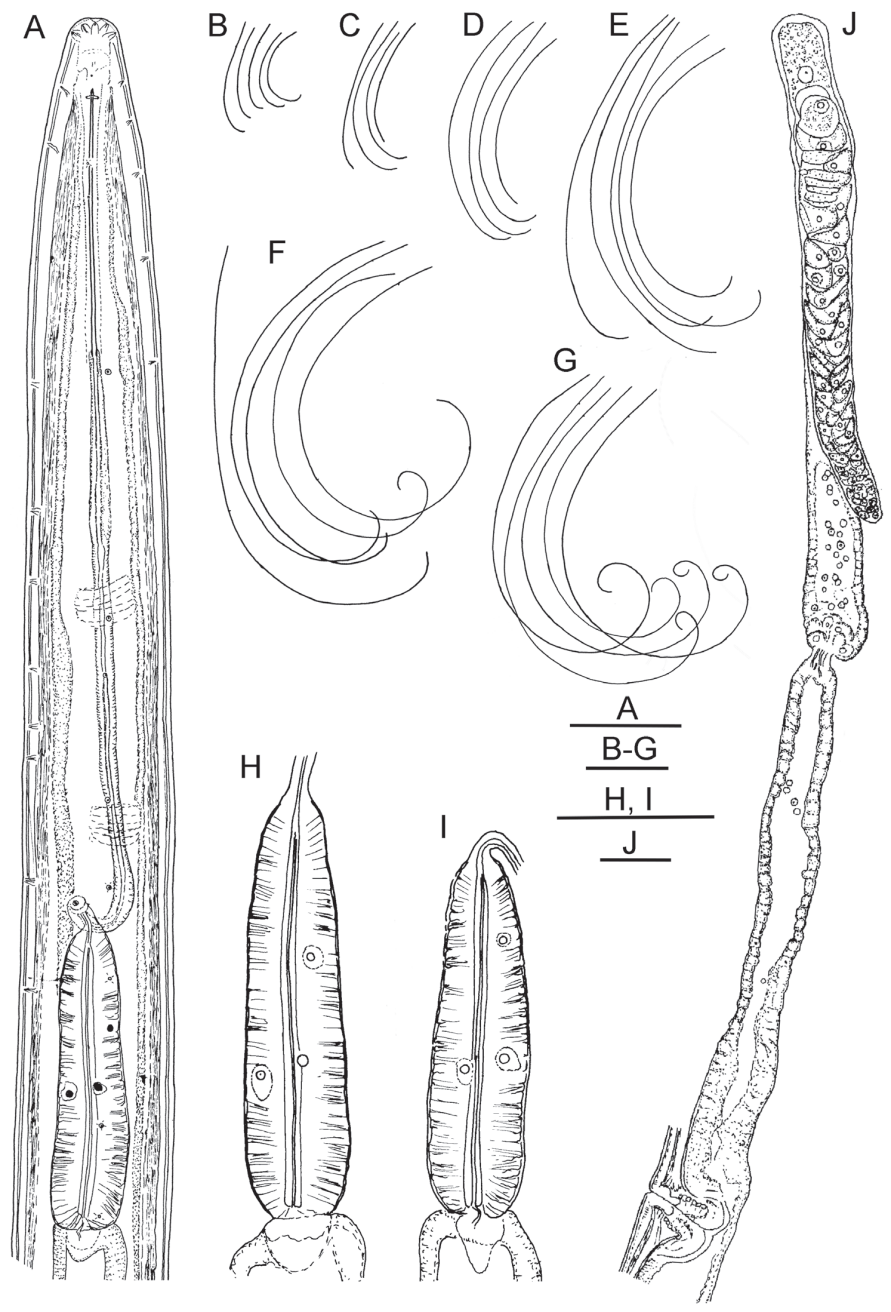
*Longidorus cholevae* sp. n. *Female*: **A** Anterior end **F** Habitus **I** Pharyngeal bulb **J** Anterior genital branch *Male*: **G** Habitus **H** Pharyngeal bulb *Juveniles*: **B–E** Habitus of first-, second-, third- and fourth-stage juveniles. Scale-bars: **A, H, I, J** 50 μm; **B–G** 1 mm.

**Figure 2.**
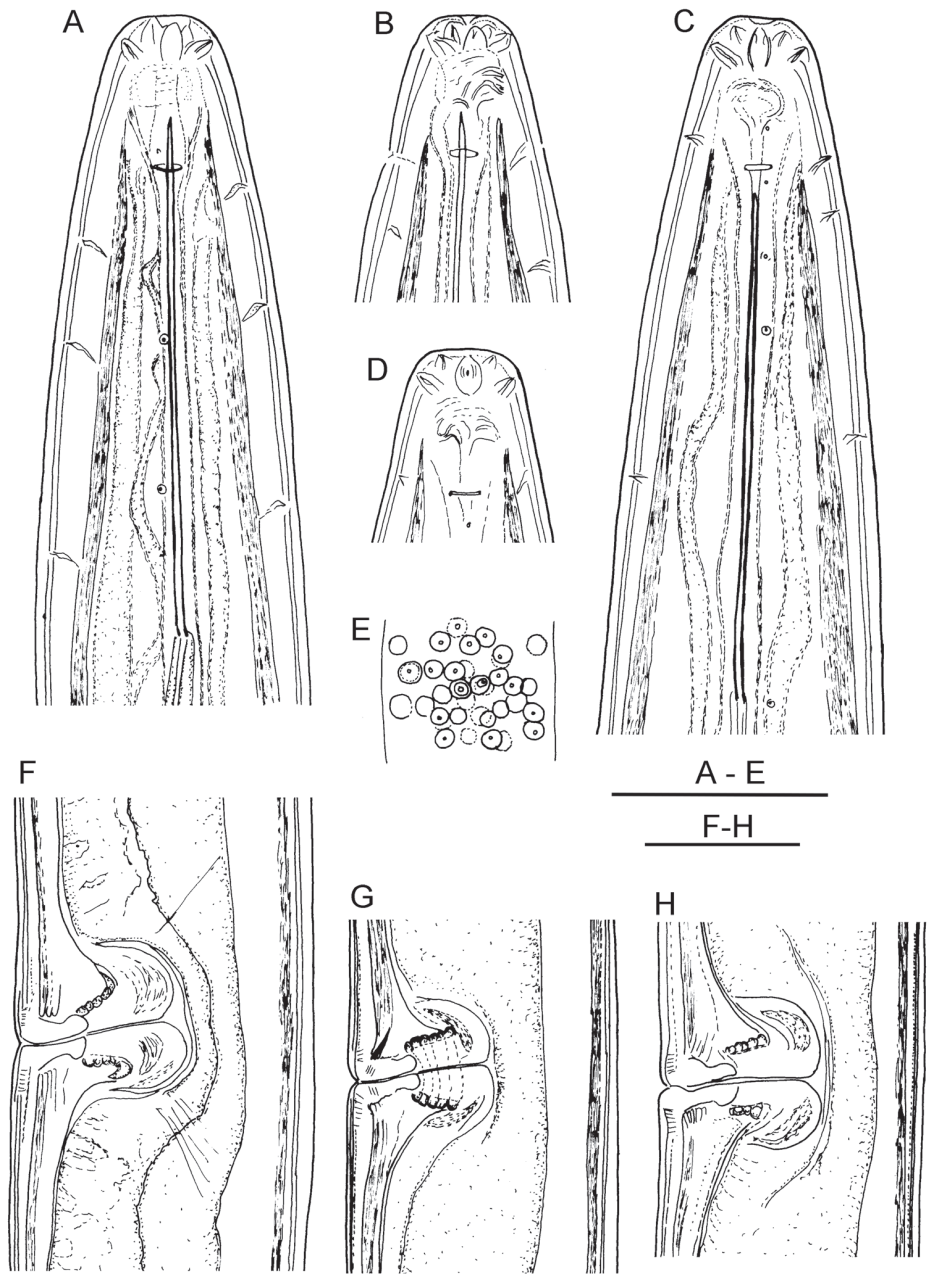
*Longidorus cholevae* sp. n. *Female*: **A** Anterior end **B** Lip region/amphidial fovea **F–H** Variations in vagina shape; *Male*: **C** Anterior end **D** Lip region/amphidial fovea **E** Sperms. Scale-bars: **A–H** 50 μm.

**Figure 3.**
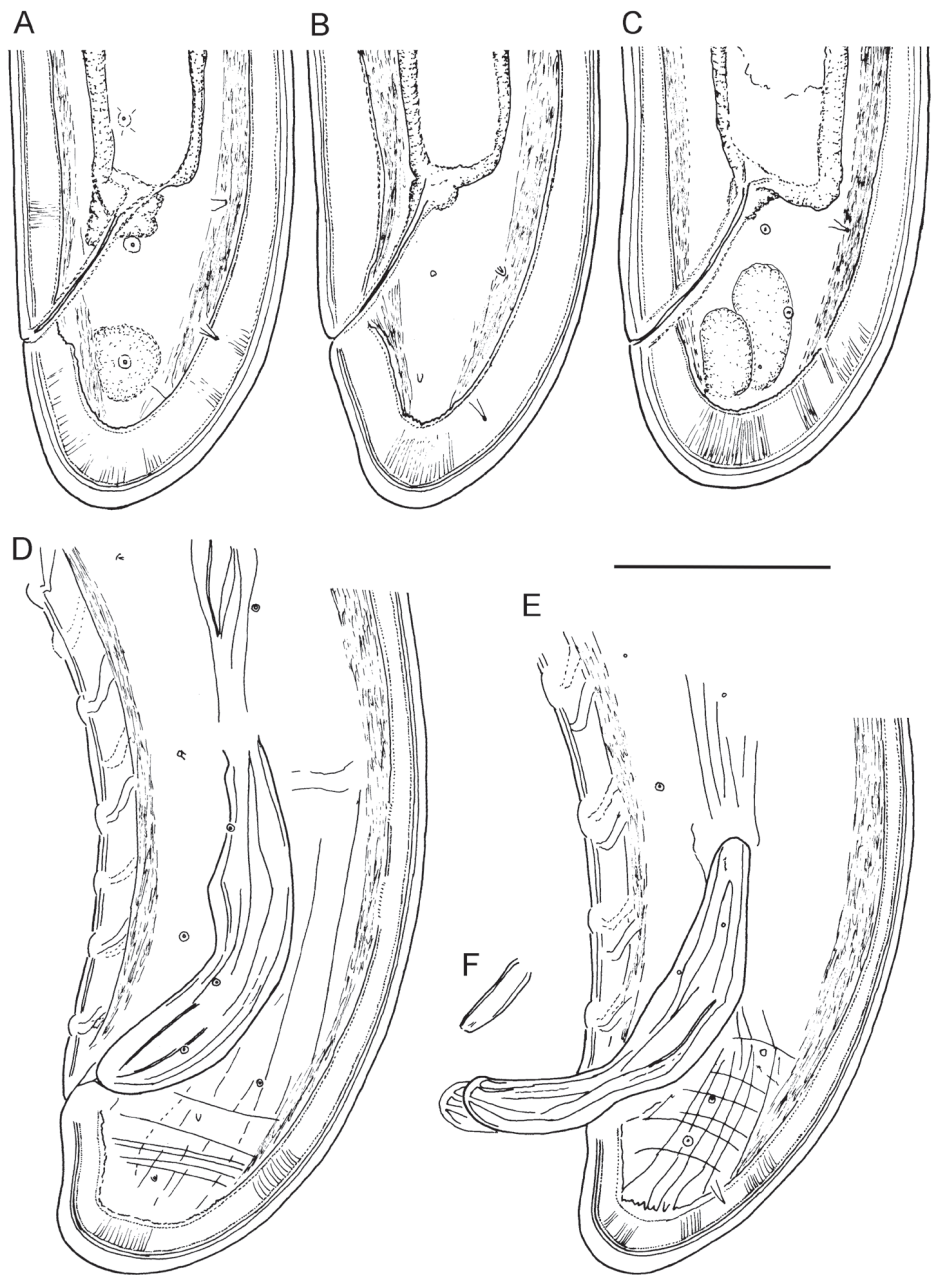
*Longidorus cholevae* sp. n. *Female*: **A–C** Variations in tail shape; *Male*: **D, E** Tail ends with spicules **F** Lateral piece. Scale-bars: 50 μm.

**Figure 4.**
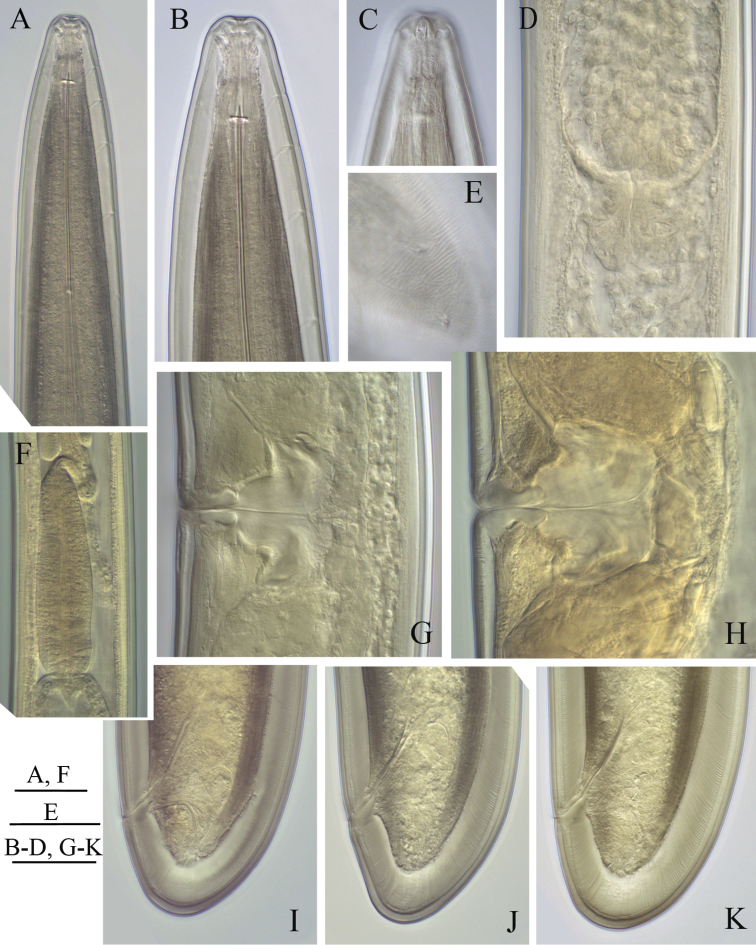
*Longidorus cholevae* sp. n. *Female*: **A** Anterior region of holotype **B** Head end **C** Amphidial fovea of holotype **D** Sphincter between uterus and *pars dilatata oviductus* **E** Caudal pores **F** Pharyngeal bulb **G, H** Variations in vagina shape; **I–K** Variations in tail shape. Scale bars: **A, F** 40 μm; **E** 20 μm; **B–D, G–K** 30 μm.

**Figure 5.**
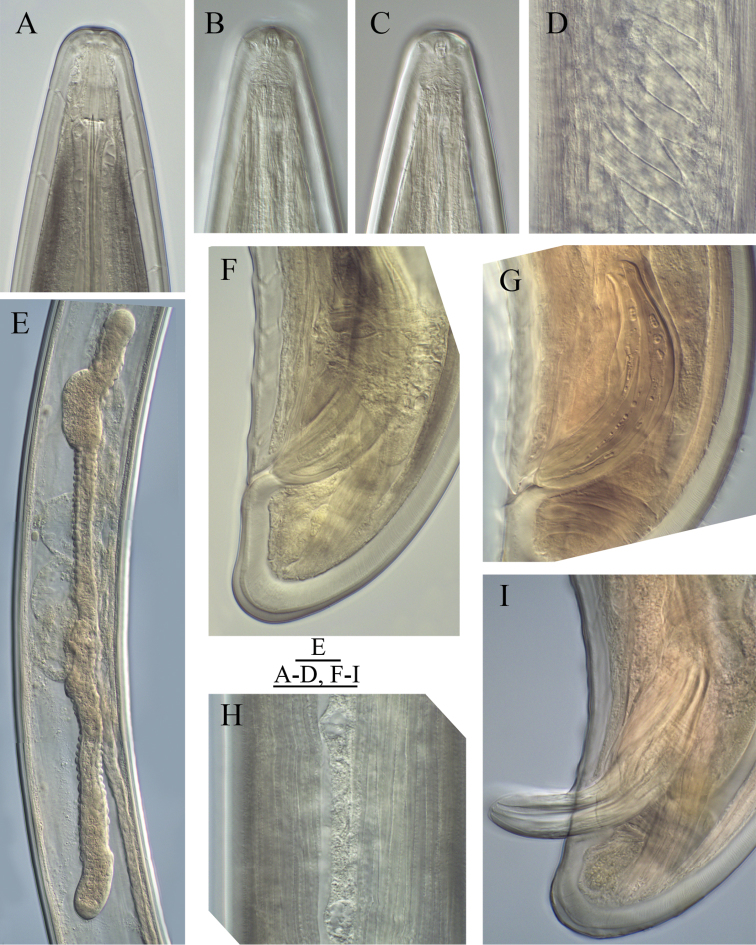
*Longidorus cholevae* sp. n. *Male*: **A** Lip region **B, C** Amphidial fovea, **B** upper view **C**, lower view **D** Part of testis with radial muscles **E** Genital system of a young male **F** Tail, **G** Spicule **H** Lateral field **I** Protracted spicule. Scale bars: **A–D, F–I** 30 µm; **E** 40 µm.

**Figure 6.**
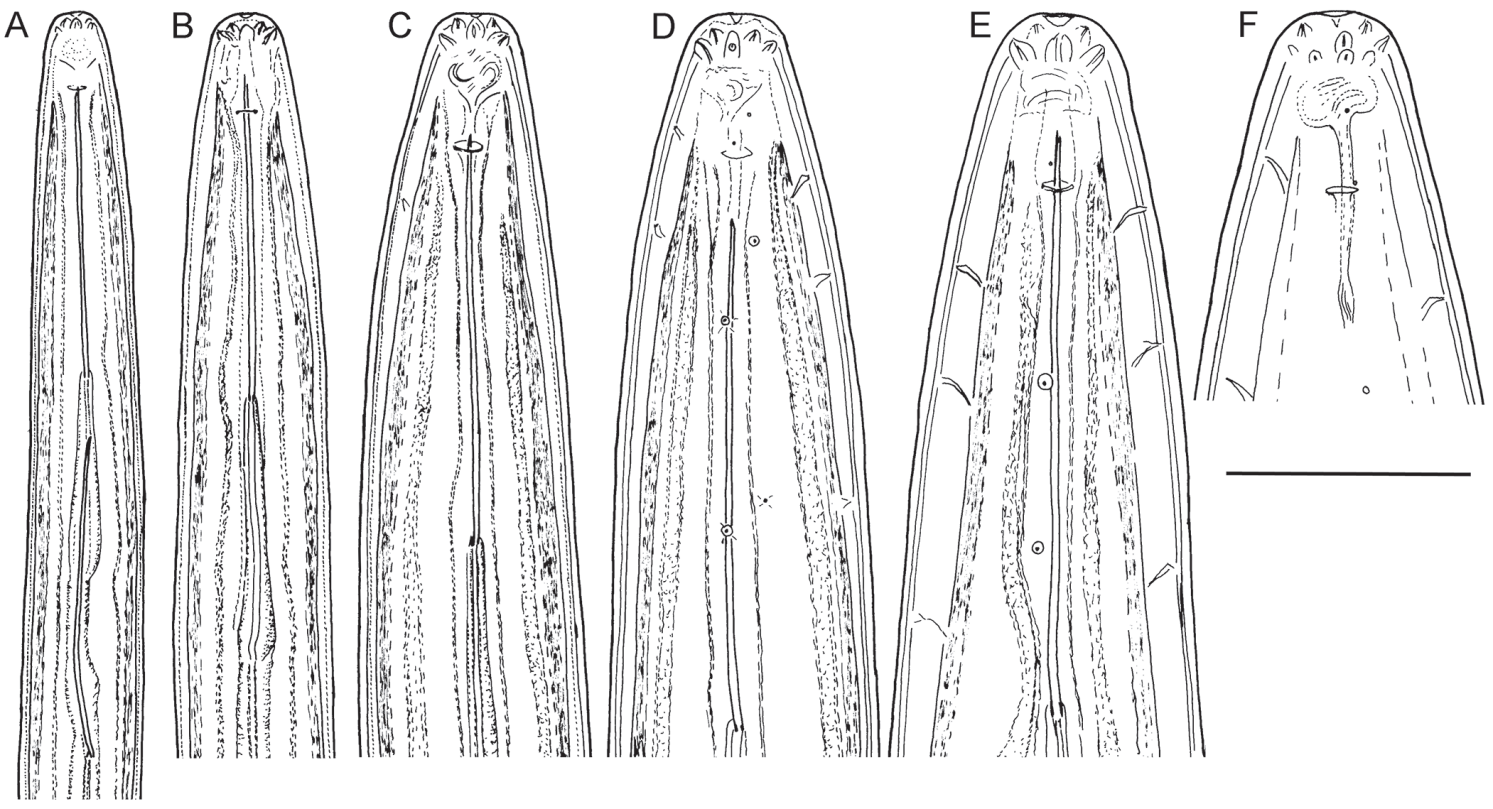
*Longidorus cholevae* sp. n. Anterior region of: **A–D** First-fourth juvenile stage **F** Female **G** Male. Scale-bar: 50 μm.

**Figure 7.**
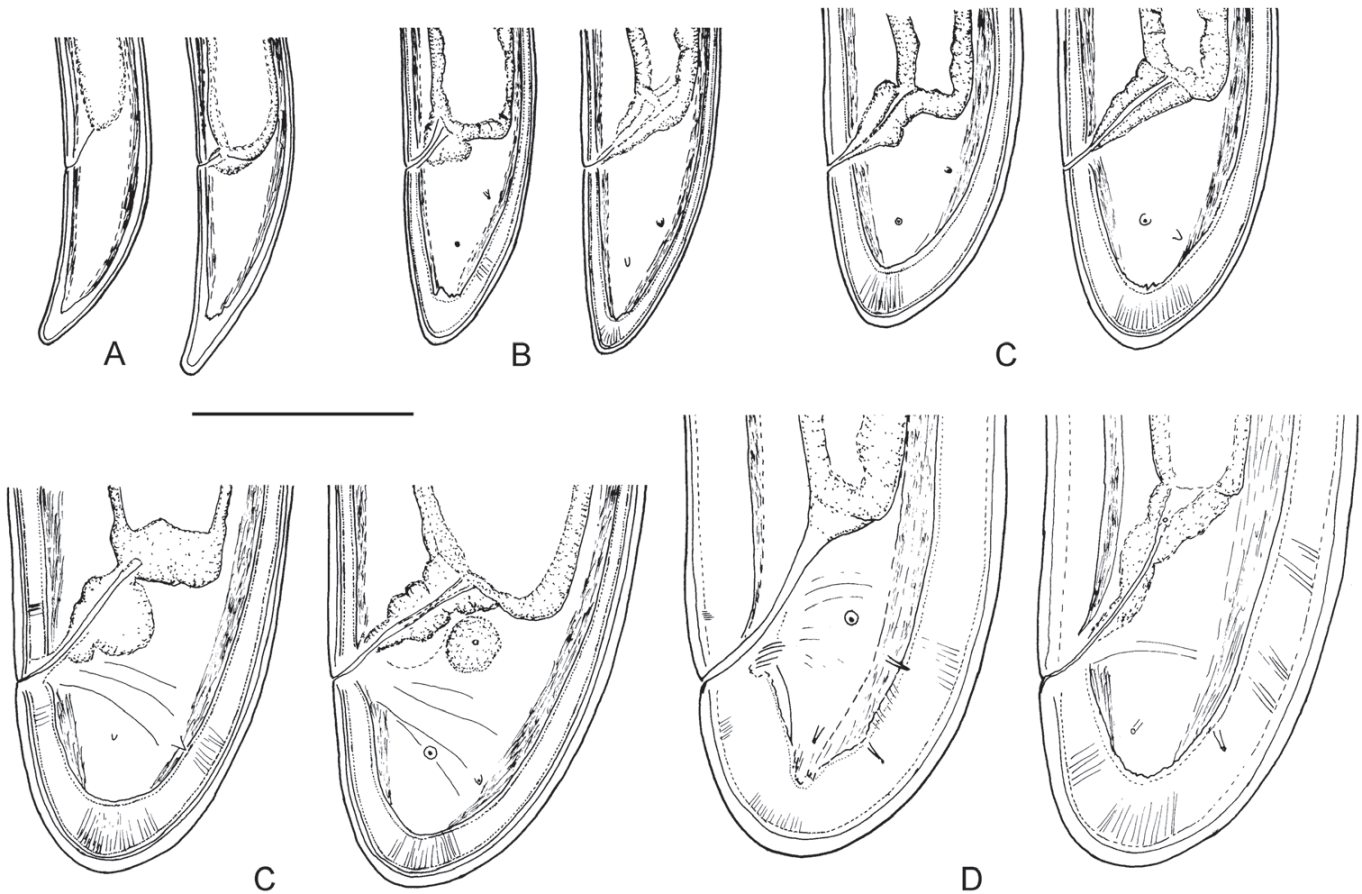
*Longidorus cholevae* sp. n. Variations in tail shape: **A–D** Tail of first-fourth juvenile stage**E** Female Scale-bar: 50 μm.

**Figure 8.**
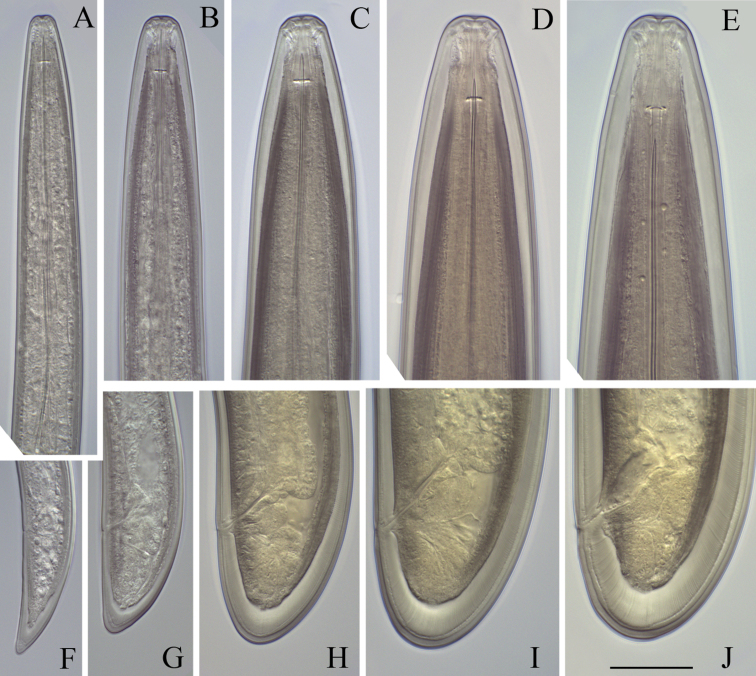
*Longidorus cholevae* sp. n. *Juveniles*: **A–D** Head ends of first- to fourth-stages **F–I** Tail end of first- to fourth-stages *Female*: **E** Anterior end **J** Tail. Scale-bar: 30 μm.

**Figure 9.**
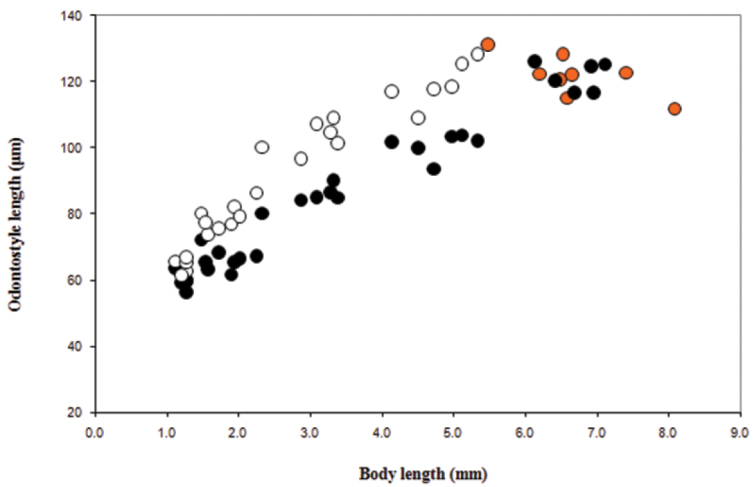
*Longidorus cholevae* sp. n. Scatter plot of the functional (˜, juveniles and adults, females in orange) and replacement (**™**, juveniles) odontostyle in relation to body length of the juvenile developmental stages and adults.

#### Type locality and plant association.

Bachevo village, Rila Mountains, co-ordinates 41°56'14.97"N, 23°25'15.02"E, 1032 m asl, riparian vegetation; soil around the roots of wild cherry (*Prunus avium* L.), *Juniperus communis* L., *Urtica dioica* L. and grasses.

#### Type material.

Holotype and 1 paratype females, 2 males, and 23 juveniles deposited in the nematode collection of the Institute of Biodiversity and Ecosystem Research, Sofia, Bulgaria. Other paratypes deposited as follows: two females, one male and 8 juveniles in the Nematode collection of the Foodand Environment Research Agency, Sand Hutton, UK (former Rothamsted Nematode Collection); one female, one male and 6 juveniles in the USDA Nematode Collection, Beltsville, Maryland, USA; one female, one male and 8 juveniles in the Riverside Nematode Collection, University of California, Riverside, USA; one female, one male and 5 juveniles in the Nematode Collection of the Institute of Plant Protection, Bari, Italy; one female, one male and 12 juveniles in the Wageningen Nematode Collection (WANECO), Wageningen, the Netherlands.

#### Diagnosis and relationships.

*Longidorus cholevae* sp. n. is a comparatively large bisexual species (6.1–8.1 mm) with odontostyle over 100 μm (106–129 μm) long, lip region wide (21.5–24 μm), continuous, anteriorly rounded, amphidial fovea pouch like, almost as wide as long, posteriorly situated guide ring, short, bluntly rounded to hemispherical tail and normal arrangement of pharyngeal glands.

The alpha-numeric codes for *Longidorus cholevae* sp. n. to be applied to the polytomic identification key for *Longidorus* species by Chen et al. (1997) are, A45, B4, C23, D1, E5, F34, G12, H1, I2. The group of comparatively large *Longidorus* species (code F34) with a long odontostyle (code A45), pouch like amphidial fovea, elongate funnel (E4) or short (funnel or stirrup shaped (E5), normal arrangement of pharyngeal glands and short rounded tail (code H1) consists of a few species: *Longidorus poessneckensis*, *Longidorus caespiticola*, *Longidorus macrosoma*, *Longidorus helveticus*, *Longidorus carniolensis*, *Longidorus macroteromucronatus* Altherr, 1974, *Longidorus pseudoelongatus* Altherr, 1976, *Longidorus pius*. It differs from all these species except for *Longidorus caespiticola* and *Longidorus pseudoelongatus*, by the more anteriorly situated guide ring (ave. 32.6 (30–37) *vs* ave. 40 (36–43) μm in *Longidorus poessneckensis*; 37–48 μm in *Longidorus macrosoma*; 37.5–48 μm in *Longidorus helveticus*; 42–47 μm in *Longidorus carniolensis*; 38 μm in *Longidorus macroteromucronatus* and ave. 38.7 (35–41) μm in *Longidorus pius*). Among the above group the new species appears most similar to *Longidorus poessneckensis* from which itdiffers by adult specimens having different shape of amphidial pouches (almost as long as wide *vs* visbly longer than wide), males abundant *vs* males rare and different tail shape in first stage juveniles (elongate conoid with narowly tapering terminus *vs* elongate conoid with blunly rounded terminus) (Sturhan and Loof 2001, Kumari et al. 2009, Lišková and Kumari 2010, Kornobis and Peneva 2011). Further, it can be differentiated from:

*Longidorus caespiticola* by females having wider (21.5–24 *vs* 16–18 μm) and differently shaped lip region (rounded *vs* smoothly rounded, almost conical), shorter tail (28.5–38 *vs* 39–47 μm) and longer spicules (96–120*vs* 88.5–93 μm), and tail in first stage juveniles (elongate conoid *vs* bluntly conoid) (Boag and Brown 1975);

*Longidorus macrosoma* by female specimens having a somewhat shorter body (L= ave. 6.8 mm (6.1–8.1) *vs* ave. 9.1 mm (6.8–12), diferently shaped lip region (rounded *vs* slightly concave) and differently shaped tail of the first stage juvenile (elongated conoid *vs* digitate) (Brown and Boag 1975);

*Longidorus helveticus* by females having different shape of amphidial fovea (almost rounded *vs* elongated), shorter odontostyle (ave. 120.1 (106–129) μm *vs* ave. 135. 4 (127–145.5) in the type population and reported range for other populations 127–142 μm, differently shaped tail in first stage juvenile (elongated conoid *vs* mucronated) and shorter hyaline portion of tail (J=10–14 *vs* J=17.5–33 μm) (Lamberti et al. 2001, Barsi and De Luca 2005, Širca and Urek 2009, Kumari and Subbotin 2012);

*Longidorus carniolensis* – by having a shorter odontostyle (106–129 *vs* 136–157 μm); males with shorter spicules (96–120 *vs* 122–145 μm); different tail shape in first stage juvenile (elongate conoid *vs* rounded, conoidal, c’=1.8–2.2 vs c’=1.2–1.5) (Širca et al. 2011);

*Longidorus macroteromucronatus* – by females having wider lip region (21.5–24 *vs* 17.5 μm (calculated from the drawing by Altherr (1974), shorter odontostyle (106–129 *vs* 133 μm) and higher c values (c=171.2–220.4 *vs* c=160);

*Longidorus pius* – by different d and d’ values (following Brown et al. 1994) (d=1.2–1.4 *vs* d=1.7–1.9; d’=1.4–1.6 *vs* d’=1.9–2.1), shorter odontostyle (ave. 120.1 (106–129 *vs* ave. 136.5 and 137.5 (128–147.5) μm), shorter tail (28.5–38 *vs* 37–46.5 μm), higher c value (c=171.2–220.4 *vs* c=114.6–166.5) in females; males abundant *vs* males rare, and different tail shape in first stage juveniles (elongate conoid *vs* subdigitate, J=10–14 *vs* J=15–20 μm) (Barsi and Lamberti 2001, Barsi and De Luca 2008). Although in the original description the code for amphidial fovea shape is D1, but in the photos it appears more like that in the new species;

*Longidorus pseudoelongatus* – by having a longer body (L=6.1–8.1 *vs* L=5.1–5.6 mm),differently shaped (continious *vs* separated by constriction) and wider lip region (21.5–24 *vs* 12 μm), higher c (c=171.2–220.4 *vs* c=100–150) and lower c’ values (c’=0.5–0.7 *vs* c’=0.93) (Altherr 1976).

Further, *Longidorus cholevae* sp. n. is similar in body and odontostyle lengths (codes F34 and A45), and shape of anterior region and tail (codes D1 and H1) with a group of several other species from which it differs in amphidial fovea shape (see Appendix 2: a partial polytomous key): *Longidorus kheirii*, *Longidorus raskii* Lamberi & Agostinelli, 1993, *Longidorus arthensis* Brown, Grunder, Hooper, Klingler & Kunz, 1994, *Longidorus fasciatus* Roca & Lamberti, 1981, *Longidorus uroshis* Krnjaić, Lamberti, Krnjaić, Agostinelli & Radicci, 2000, *Longidorus silvae* Roca, 1993, *Longidorus iuglandis* Roca, Lamberti & Agostinelli, 1984, *Longidorus saginus* Khan, Seshardi, Weischer & Mathen, 1971, *Longidorus picenus* Roca, Lamberti & Agostinelli, 1984, *Longidorus baeticus*). The new species can be distinguished from *Longidorus raskii*, *Longidorus arthensis*, *Longidorus fasciatus*, *Longidorus uroshis*, *Longidorus silvae*, *Longidorus picenus* and *Longidorus baeticus* by its wider lip region (21.5–24 μm *vs* 15–19 μm; 14–19 μm; 12–14 μm; 14–20.5; 14–17 μm; 14–16 μm; 19–22 μm; 12–14.5 μm); from *Longidorus raskii*, *Longidorus uroshis* and *Longidorus saginus* by having different odontostyle length (106–129 μm *vs* 76–103 μm; 120–152 μm and 135–155 μm); from *Longidorus kheirii*, *Longidorus raskii*, *Longidorus arthensis* and *Longidorus uroshis* by the shorter tail (28.5–38 *vs* 47–72 μm; 36–50 μm; 36–46.5 μm and 38–57 μm); from *Longidorus kheirii*, *Longidorus silvae* and *Longidorus picenus* by having more anteriorly situated guide ring (30–37 *vs* 36.5–45 μm, 36–48 μm and 37–42 μm). Additionally, it can be differentiated from:

*Longidorus kheirii* by females having differently shaped lip region (rounded *vs* slightly concave), higher c value (171.2–220.4 *vs* 119–167.8), smaller pharyngeal bulb (114.5–146.5 × 30–38 *vs* 149.5–193.5 × 39.5–48 μm), males abundant, functional *vs* rare and not functional, differently shaped tail of the first stage juvenile as well as different morphometrics concerning the main characters such as body and tail length, functional and replacement odontostyle length (Table 1; Table 2 in Pedram et al. 2008).

*Longidorus raskii* by females having different tail shape in first stage juveniles (elongate conoid *vs* bluntly conoid); (Lamberti et al. 2001, Krnjaić et al. 2002, Barsi and De Luca 2005);

*Longidorus arthensis* by females having lower c’ value (c’=0.5–0.7 *vs* c’=0.8–1.1 and 0.9–1.1); males with longer spicules (96–120 vs 60–66 μm); different tail shape in first stage juveniles (elongate conoid *vs* digitate) (Brown et al. 1994, Lamberti et al. 2001);

*Longidorus fasciatus* by females having a more plump body (a=61.1–83.3 *vs* a=121–143) (Roca and Lamberti 1981);

*Longidorus uroshis* by males with longer spicules (96–120 *vs* 59–72 and 64–78 μm) and different tail shape in first stage juveniles (elongate conoid *vs* digitated) (Krnjaić et al. 2000, Krnjaić et al. 2002, Sturhan and Lišková 2002);

*Longidorus silvae* by female specimens having differently shaped lip region (rounded *vs* sub-acute and flattened anteriorly) and tail of the first and second stage juvenile (elongated conoid *vs* mucronated; conoid *vs* bluntly rounded, respectively), and males abundant *vs* males rare (Roca 1993, Barsi and Lamberti 2004, Barsi et al. 2007).

*Longidorus iuglandis* by having longer uteri (357.5–662.5 *vs* 140–160 μm) and differently shaped tail in the first stage juvenile (elongate conoid *vs* bluntly rounded) (Roca et al. 1984);

*Longidorus saginus* by having a longer body (L=6.1–8.1 *vs* 4.8–6.4 mm); lower c’ value (c’’=0.5–07 *vs* c’=0.8); more posteriorly situated vulva (V=46.7–53.4 *vs* V=40–45) (Khan et al. 1971);

*Longidorus picenus* by having, differently shaped tail in the first stage juvenile (elongate conoid *vs* mucronated) (Roca et al. 1985);

*Longidorus baeticus* by males having longer spicules (96–120 *vs* 80–95 μm) and differently shaped tail in the first stage juvenile (elongate conoid *vs* bluntly rounded to cylindrical).

#### Etymology.

The species is named after Dr Boryana Choleva, Faculty of Biology, University of Sofia, retired, for her substantial contribution to the knowledge of the fam. Longidoridae in Bulgaria.

##### Phylogenetic relationships of *Longidorus cholevae* with other *Longidorus* species

The amplification of D2-D3 expansion domains of the 28S rDNA and the ITS containing region yielded single fragments of 800 bp and 1384 bp, respectively, based on sequencing. The ITS1 and ITS2 sizes were 579 bp and 338 bp, respectively that resulted in the shortest ITS recorded for *Longidorus* so far. Intra-individual and intra-population sequence variability in ITS and no variability in D2D3 domains have been observed.

A BLAST search for D2-D3 region showed a 80-93% degree of similarity among *Longidorus* spp. suggesting that *Longidorus cholevae* can be easily identified from other species by using this ribosomal region. The closest species were *Longidorus poessneckensis* (93% similarity), *Longidorus caespiticola*, *Longidorus macrosoma* and *Longidorus helveticus* (92% similarity). Pairwise BLAST comparisons of the ITS sequence of *Longidorus cholevae* with those of *Longidorus* spp. from the database displayed high nucleotide dissimilarity and considerable variation in length.

Our preliminary phylogenetic analyses based on all the D2-D3 *Longidorus* sequences deposited in NCBI revealed that the new species clusters into a well-supported group of *Longidorus* species having a European distribution: *Longidorus caespiticola*, *Longidorus macrososma*, *Longidorus poessneckensis*, *Longidorus helveticus* and *Longidorus carniolensis* (trees not presented). The monophyly of this group has been highly supported also in other studies, including SSU phylogenetic analyses (Robbins et al. 2009, Gutiérrez-Gutiérrez et al. 2013). All these are large species, very similar in their morphology having long odontostyles, elongated or short not bilobed pouch-like amphidial fovea, continuous head region, short bluntly conoid to almost hemisphaercial tail, mainly amphimictic (only with *Longidorus macrosoma* and *Longidorus poessneckensis* males are rare). *Longidorus caespiticola* and *Longidorus macrososma* occur mainly in western Europe including the British Isles, *Longidorus poessneckensis* was reported from central (Germany, Slovakia and Czech Republik) and northern Europe (Poland); the first two species were found in association with a wide range of crops and forest trees (Brown and Boag 1975, Boag and Brown 1975); *Longidorus poessneckensis* with preference toflood plains and hill deciduos forest habitat (Lišková and Kumari 2010) and *Longidorus helveticus* associated with deciduous forest and orchard threes in central Europe (Lamberti et al. 2001, Širca and Urek 2009, Kumari and Subbotin 2012). *Longidorus carniolensis* is known only from Slovenia (grapevine) and *Longidorus cholevae* sp. n. – only from Bulgaria (riparian vegetation). Probably, *Longidorus pius*, known so far only from Macedonia and having similar morphology, is part of this group, however, no sequences of D2-D3 region are available.

**Figure 10.**
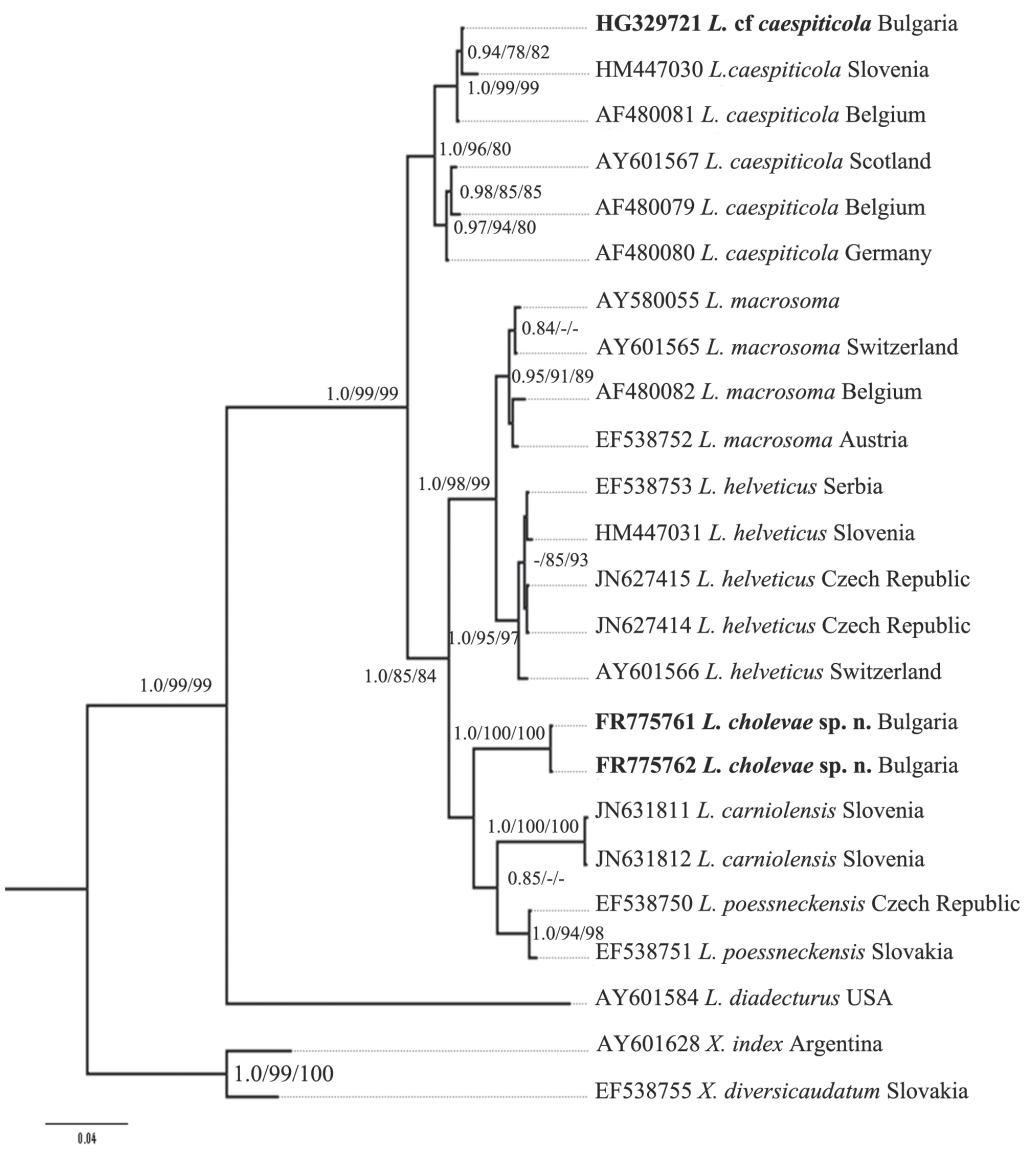
Phylogenetic relationships of *Longidorus cholevae* sp. n. and its closest species for the D2-D3 rDNA. Bayesian Inference strict consensus tree acquired under GTR+G model. Numbers at the nodes indicating posterior probabilities higher that 0.8 and bootstrap values more that 70% for ML and NJ are presented.

**Figure 11.**
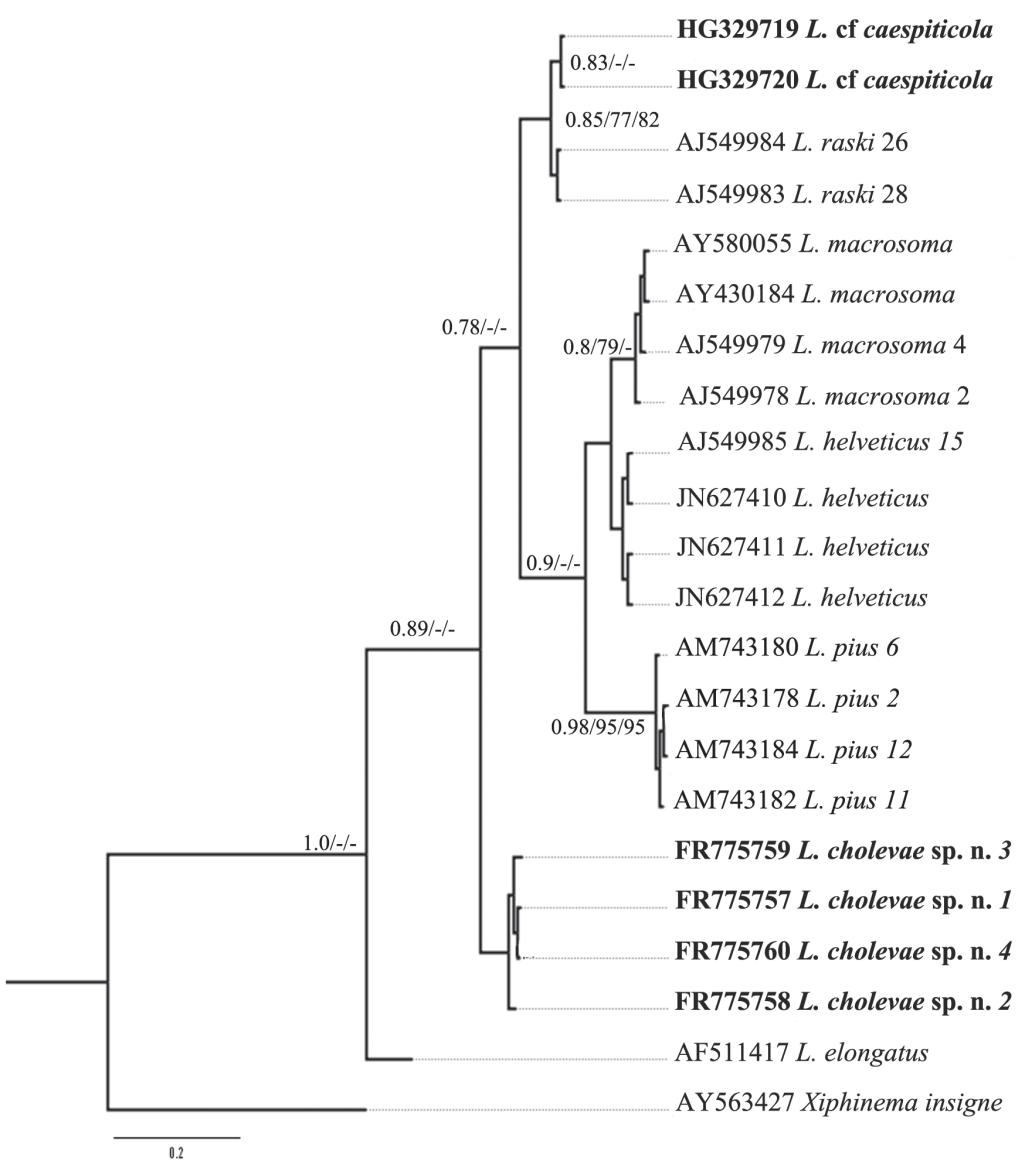
Phylogenetic relationships of *Longidorus cholevae* sp. n. and its closest species for the partial 18S-ITS1 rDNA regions. Bayesian Inference strict consensus tree acquired under K2+G model. Numbers at the nodes indicating posterior probabilities higher that 0.8 and bootstrap values more that 70% for ML and NJ are presented.

Further, for phylogenetic analysis *Longidorus* species from GenBank with the highest match of BLAST search were aligned along with *Longidorus cholevae* D2-D3 and partial 18S-ITS1 sequences and these alignments included sequences from various populations (Table 1). The trees obtained by NJ, ML and BI methods showed similar topology and differed in the position of poorly supported clades, and thus only the BI trees with posterior probabilities higher than 0.8 and bootstrap values above 70% (NJ and ML) are presented (Figs 10–11).

The phylogenetic tree of the D2-D3 region (Fig. 10) showed two well-supported clades: Clade I consists of three subclades: two highly supported subclades containing various populations of I1) *Longidorus helveticus* and I2) *Longidorus macrosoma*, and one subclade having lower values for ML bootstrap support (52%) and BI posterior probabilities (0.72) I3) that includes the new species *Longidorus cholevae*, two populations of *Longidorus carniolensis* from Slovenia and two populations of *Longidorus poessneckensis* from the Czech Republic and Slovakia. The second clade (II) consists of two well-supported subclades: II1) consisted of *Longidorus caespiticola* from Slovenia and Belgium and one *Longidorus* cf. *caespiticola* from Bulgaria and subclade II2) consisted of three populations of *Longidorus caespiticola* from Scotland, Belgium and Germany. It is possible that these populations represent two different species that requires further investigation.

The phylogenetic reconstructions of the partial 18S-ITS1 region revealed more unstable groups due to the shorter sequence length and higher sequence variability. Three of the *Longidorus* spp. belonging to the above mentioned group (*Longidorus* cf. *caespiticola*, *Longidorus helveticus* and *Longidorus macrosoma*)and two additional species(*Longidorus pius* and *Longidorus raskii*) originating from Macedonia and Switzerland have been separated from other ITS1 *Longidorus* sequences (the tree not presented) and further analysed (Fig. 11). Three clades were distinguished, two well supported clades consisting of: 1) *Longidorus macrosoma*, *Longidorus helveticus* and *Longidorus pius* and 2) *Longidorus* cf. *caespiticola* and *Longidorus raskii*, and one not well resolved 3) containing only *Longidorus cholevae* sp. n. The species forming these clades have similar tail shape in first stage juveniles: digitate in clade 1, bluntly conoidal in clade 2, elongate conoidal in clade 3.

## Supplementary Material

XML Treatment for Longidorus cholevae

## Appendix 1

List of the species of the genus *Longidorus* (doi: 10.3897/zookeys.330.5750.app1) File format: Microsoft Word Document (doc).

**Explanation note:** List of the species of the genus *Longidorus* Micoletzky, 1922.

## Appendix 2

A partial polytomous key to the species of *Longidorus* (doi: 10.3897/zookeys.330.5750.app2) File format: Microsoft Word Document (doc).

**Explanation note:** A partial polytomous key to the species of *Longidorus* with long odontostyle (A45) and short tail (H1) based on the key by Chen et al. (1997) incorporating species described after 1997 and those transferred from other genera, see Appendix 1.
