# Supplementary material for: Description of Longidorus cholevae sp. n. (Nematoda, Dorylaimida) from a riparian habitat in the Rila Mountains, Bulgaria
Source: Zookeys. 2013 Sep 9;(330):1–26. doi: 10.3897/zookeys.330.5750 (PMC3800803; doi:10.3897/zookeys.330.5750)
Supplement: Supplementary file 2 — List of the species of the genus Longidorus (doi: 10.3897/zookeys.330.5750.app1) File format: Microsoft Word Document (doc). [file ZooKeys-330-001-s001.doc]

List

of the species of the genus *Longidorus* Micoletzky, 1922

Type species *Longidorus elongatus* (de Man, 1876) Micoletzky, 1922

*Longidorus aetnaeus* Roca, Lamberti, Agostinelli & Vinciguerra, 1986 – Italy, Sicily, *Quercus ilex* L.

*Longidorus africanus* Merny, 1966 – Rhodesia, *Saccarum* *officinarum* L.

*Longidorus alaskaensis* Robbins & Brown, 1996 – USA, Alaska, willow, rose, aspen

*Longidorus alvegus* Roca, Pereira & Lamberti, 1989 – Portugal, *Capsicum annum* L.

*Longidorus* *americanus* Handoo, Carta, Skantar, Ye, Robbins, Subbotin, Fraedrich, Stephen & Cram, 2005 – USA, Georgia, *Pinus taeda* L.

*Longidorus ampullatus* Jacobs & Heyns, 1987 – South Africa, wild *Ficus* sp.

*Longidorus* *andalusicus* Gutiérrez- Gutiérrez, Cantalapiedra-Navarrete, Monte-Borrego, Palomares-Rius & Castillo, 2013 – Spain, fallow soil

*Longidorus apuloides* Roca, 1996 – Italy, *Populus nigra* L.

*Longidorus apulus* Roca & Bleve-Zacheo, 1977 – Italy, artichoke, potato, chicory, fennel, weeds

*Longidorus arenosus* Kankina & Ivanova, 1986 – Tadzhikistan, *Calligonum* *mucrocarpum* Borzcz., *Haloxylon* *persicum* Bunge, *Populus* *pruinosa* Schrenk.

*Longidorus arthemisiae* Rubtsova, Chizhov & Subbotin, 1999 – European part of Russia, *Artemisia* sp.

*Longidorus arthensis* Brown, Grunder, Hooper, Klinger & Kunz, 1994 – Switzerland, cherry tree

*Longidorus athesinus* Lamberti, Coiro & Agostinelli, 1991 – Italy, cherry tree

*Longidorus attenuatus* Hooper, 1961 – England, lettuce

*Longidorus auratus* Jacobs & Heyns, 1987 – South Africa, *Vitis vinifera* L.

*Longidorus baeticus* Gutiérrez- Gutiérrez, Cantalapiedra-Navarrete, Monte-Borrego, Palomares-Rius & Castillo, 2013 – Spain, *V. vinifera*

*Longidorus balticus* Brzeski, Peneva & Brown, 2000 – Poland, sand dunes, *Elymus* *arenarius* L., *Salix* sp.

*Longidorus belloi* Fe Andrés & Arias, 1988 – Spain, permanent pasture

*Longidorus belondiroides* Heyns, 1966 *–* South Africa, dark clay soil

*Longidorus bernardi* Robbins & Brown, 1996 – USA, Alaska, willow, rose, aspen

*Longidorus biformis* Ye & Robbins, 2004 – USA, Arkansas, *Ulmus americana* L., *Celtis occidentalis* L., *Acer* sp*.*

*Longidorus boshi* (Khan, Chawla & Saha, 1972) Decraemer & Coomans 2007 – India, *Prunus* *domestica* L.

*Longidorus breviannulatus* Norton & Hoffmann, 1975 – USA, Iowa, *Zea mays* L.

*Longidorus brevis* Swart, Cadet & N’Diaye, 1996 – Senegal, *Guiera senegalensis* J. F. Gmelin, *Pennisetum pedicellatum* Trin., *Cordylla pinnata* (A. Rich.), *Blumea aurita* (L.F.) DC

*Longidorus caespiticola* Hooper, 1961 – England, permanent turf along the road

*Longidorus camelliae* Zheng, Peneva & Brown, 2000 – China, *Camellia japonica* L.

*Longidorus carniolensis* Širca, Urek, Lasarova, Elshishka & Peneva, 2011 *–* Slovenia, *V. vinifera*

*Longidorus carpathicus* Lišková*,* Robbins & Brown, 1997 – Slovakia, *Fagus* *sylvatica* L.

*Longidorus carpetanensis* Arias, Andres & Navas, 1986 – Spain, *Cytisus purgans* L.

*Longidorus cedari* (Siddiqi, 1976) Decraemer et Coomans, 2007 – India, Uttar Pradesh, *Cedrus* *libani* A. Rich.

*Longidorus closelongatus* Stoyanov, 1964 – Bulgaria, *V. vinifera*

*Longidorus cohni* Heyns, 1969 – Israel, alfalfa, Rhode grass

*Longidorus concavus* Singh & Khan, 1997 – India, *Prunus domestica* L.

*Longidorus congoensis* Aboul-Eid, 1970 – Congo, *Arundinaria alpina* K. Schum. in a bamboo forest

*Longidorus conicaudatus* Khan, 1986 – India, *Thuja* sp.

*Longidorus conicaudoides* Jacobs & Heyns, 1987 – South Africa, pineapples

*Longidorus conicephalus* Singh & Khan, 1997 – India, *Musa paradisiaca* L.

*Longidorus crassus* Thorne, 1974 – USA, South Dakota, native sod

*Longidorus crataegi* Roca & Bravo, 1996 – Portugal, *Crataegus oxycantha* L.

*Longidorus cretensis* Tzortzakakis, Peneva, Terzakis, Neilson & Brown, 2001 – Crete, Greece, *V. vinifera*

*Longidorus curvatus* Khan, 1986 – India, *Pinus longifolia* Roxb.

*Longidorus cylindricapitatus* Krnjaić, Roca, Krnjaić & Agostinelli, 2005 – Serbia, *Picea omorika* (Pancic) Purkyne

*Longidorus cylindricaudatus* Kozlowska & Seinhorst, 1979 – The Netherlands, Germany, *Pinus sylvestris* L.*, Quercus robur* L.*, Calluna vulgaris* (L.) Hull.

*Longidorus dalmassoi* Peneva, Loof & Brown, 1998 – France, meadow grasses

*Longidorus danuvii* [Barsi,](http://apps.isiknowledge.com/WoS/CIW.cgi?SID=4FGi9eE9C6JkFEGmCND&Func=OneClickSearch&field=AU&val=Barsi+L&ut=000249098200010&auloc=1&fullauth= (Barsi, Laszlo)&curr_doc=132/1&Form=FullRecordPage&doc=132/1) [Lamberti &](http://apps.isiknowledge.com/WoS/CIW.cgi?SID=4FGi9eE9C6JkFEGmCND&Func=OneClickSearch&field=AU&val=Lamberti+F&ut=000249098200010&auloc=2&fullauth= (Lamberti, Franco)&curr_doc=132/1&Form=FullRecordPage&doc=132/1) [De Luca,](http://apps.isiknowledge.com/WoS/CIW.cgi?SID=4FGi9eE9C6JkFEGmCND&Func=OneClickSearch&field=AU&val=De+Luca+F&ut=000249098200010&auloc=3&fullauth= (De Luca, Francesca)&curr_doc=132/1&Form=FullRecordPage&doc=132/1) 2007 – Serbia, *Amorpha* *fruticosa* L., other population around roots of *Populus* sp.

*Longidorus diadecturus* Eveleigh & Allen, 1982 – Canada, Ontario, peach

*Longidorus* *dimorphicaudatus* Baniyamuddin & Ahmad, 2006 – India, grasses

*Longidorus distinctus* Lamberti, Choleva & Agostinelli, 1983 – Bulgaria, *Juglans regia* L., *Cydonia oblonga* Mill.

*Longidorus doonensis* (Singh & Khan, 1996) Ye & Robbins, 2004– India, *Prunus armeniaca* L.

*Longidorus dunensis* Brinkmam, Loof & Barbez, 1987 – the Netherlands, *Hippophae rhamnoides* L. (sea buckthorn)

*Longidorus edmundsi* Hunt & Siddiqi, 1977 – St Lucia Island, West Indies, beach, *Coccoloba* *uvifera* L. (sea grape)

*Longidorus elongatus* (de Man, 1876) Micoletzky, 1922 – The Netherlands, grass

*Longidorus eridanicus* Roca, Lamberti & Agostinelli, 1984 – Italy, grass and woodlands, *V. vinifera*

*Longidorus euonymus* Mali & Hooper, 1974 – Czech Republic, *Euonymus* *europaeus* L. (spindle tree)

*Longidorus fagi* Peneva, Choleva & Nedelchev, 1997 – Bulgaria, *Fagus sylvatica* L.

*Longidorus fangi* Xu & Cheng, 1991 – China, *Malus* *pumila* Mill.

*Longidorus fasciatus* Roca & Lamberti, 1981 – Greece, Italy, Sicily, *Cynara* *cardunculus* v. *scolymus* L. (artichoke)

*Longidorus ferrisi* Robbins, Ye et Pedram, 2009 – USA, California, Mandarin orange

*Longidorus fragilis* Thorne, 1974 – USA, Minnesota, river bank soil

*Longidorus fursti* Heyns, Coomans, Hutsebaut & Swart, 1987 – S Africa, *Cynodon* *dactylon* (L.) Pers., *Mesembryanthemum aitonis* Jacq., *Chrysanthemoides monilifera* (L.) Norl., *Acacia cyclops* A. Cunn. ex G. Don

*Longidorus globulicauda* Dalmasso, 1969, France, river bank

*Longidorus glycines* Ye & Robbins, 2004 – USA, Arkansas*, Glycine max* L. (Merrill.)

*Longidorus goodeyi* Hooper, 1961 – England, permanent turf along the road

*Longidorus grandis* Ye & Robbins, 2003 – USA, Arkansas, *Ulmus americana* L., *Maclura pomifera* (Raf. Schneid.), *Platanus occidentalis* L., *Salix* sp.

*Longidorus hangzhouensis* Zheng, Peng, Robbins & Brown, 2001 – China, *Osmanthus delavayi* L. in a botanical garden

*Longidorus helveticus* Lamberti, Kunz, Grunder, Molinari, De Luca, Agostinelli & Radicci, 2001 – Switzerland, *Prunus avium* L.

*Longidorus henanus* Xu & Cheng, 1992 – China, *V. vinifera*

*Longidorus heynsi* Andrassy, 1970 – South Africa, river Vaal

*Longidorus himalayensis* (Khan, 1986) Xu & Hooper, 1990 – India, *Prunus percica* L.

*Longidorus holovachovi* Peneva, Susulovsky et Lazarova, 2009 – Ukraine, *F. sylvatica* (beech forest)

*Longidorus igoris* Krnjaić, Lamberti, Krnjaić, Agostinelli & Radicci, 2000 – Montenegro, *Crithmum maritimum* L., *Smilax aspera* L.

*Longidorus indicus* Prabha, 1973 – India, *Moringa pterigosperma* Gaertn.

*Longidorus intermedius* Kozlowska & Seinhorst, 1979 – The Netherlands, Germany – *Prunus* *serotina* Ehrh., *Q.* *robur* L., *C.* *oxycantha* L.

*Longidorus iranicus* Surhan & Barooti, 1983 – Iran*, V. vinifera*

*Longidorus ishigakiensis* Hirata, 2002 – Japan, Okinawa, *Pleioblastus* *linearis* (Hack.)

*Longidorus ishrati* Javed, 1983 – India, *Tamarindus indiscus* L.

*Longidorus israelensis* Peneva, Orion, Shlevin, Bar-Eyal & Brown, 1998 – Israel, *Daucus* *carota* L.

*Longidorus iuglandis* Roca, Lamberti & Agostinelli, 1984 – Italy, *J. regia*

*Longidorus jagerae* Heyns & Swart, 1998 – S. Africa, *Galenia* *africana* L.

*Longidorus jiangsuensis* Xu & Hooper, 1990 – China, *Fragaria* x *ananassa* Duch.

*Longidorus jonesi* Siddiqi, 1962 – India, *P. armeniaca*

*Longidorus juglandicola* Liskova, Robbins & Brown, 1997 – Slovakia, *J. regia*

*Longidorus juvenilis* Dalmasso, 1969 – France, alluvial soil

*Longidorus juveniloides* Jacobs & Heyns, 1987 – South Africa, grass

*Longidorus kakamus* Jacobs & Heyns, 1987 – South Africa, *V. vinifera*

*Longidorus kheirii* Pedram, Niknam, Robbins Ye & Karegar, 2008 – Iran, *Rosa* sp.

*Longidorus kuiperi* Brinkmam, Loof & Barbez, 1987 – the Netherlands, France, Italy, *Ammophila arenaria*  Link. (marram grass)

*Longidorus laevicapitatus* Williams, 1959 – Mauritius, sugar cane

*Longidorus laricis* Hirata, 1995 – Japan, *Larix kaempferi* (Lamb.)

*Longidorus leptocephalus* Hooper, 1961 – England, permanent grassland

*Longidorus lignosus* Chizhov, Subbotin, Romanenko & Kruchina, 1991 – Georgia, Abhasia, *Rhododendron* sp., *Quercus iberica* Stev.

*Longidorus litchii* Xu & Cheng, 1992 – China, *Litchi chinensis* Sonn.

*Longidorus longicaudatus* Siddiqi, 1962 – USA, South Carolina, unknown host

*Longidorus lusitanicus* Macara, 1985 – Portugal, *Populus* x *euroamericana* (Dode)

*Longidorus macromucronatus* Siddiqi, 1962 – India, *Malus sylvestris* (L.) Mill.

*Longidorus macrosoma* Hooper, 1961 – England, established woodland

*Longidorus macroteromucronatus* Altherr, 1974 – Germany, ground water

*Longidorus magnus* Lamberti, Bleve-Zacheo et Arias, 1982 - Malta, *Prunus* *domestica*, L. *Vitis* sp.

*Longidorus major* Roca & D’Errico, 1987 *–* Italy, *Vitis* sp.

*Longidorus makatinus* Jacobs & Heyns, 1987 – South Africa, vineyards, sugar cane

*Longidorus martini* Merny, 1966 – Rodesia, *Vitis* sp.

*Longidorus milanis* (Krnjaić, Lamberti, Krnjaić, Agostinelli et Radicci, 2000) Roca, 2006 – Montenegro, *Quercus* *pubescens* Wild

*Longidorus mindanaoensis* Coomans, Tandingan De Ley, Angsinco Jimenez & De Ley, 2012 – Philippine, *Avicennia* mangrove habitat

*Longidorus mirus* Khan, Chawla & Seshardi, 1971 *–* India, *Citrus limon* Burm., *Punica granatum* L.

*Longidorus mobae* Jacobs & Heyns, 1987 – S Africa, sugar cane

*Longidorus moesicus* Lamberti, Choleva & Agostinelli, 1983 – Bulgaria, *Ribes nigrum* L.

*Longidorus monegrensis* (Escuer et Arias, 1997) Decraemer & Coomans, 2007 – Spain, *Rhamno-Cocciferetum*

*Longidorus monile* Heyns, 1966 – S. Africa, *Themeda triandra* Forsk. (virgin grass)

*Longidorus moniloides* Heyns, 1966 – S. Africa, *T. triandra*, *Acacia* sp.

*Longidorus naganensis* Hirata, 1995 – Japan, *Fagus crenata* Blume

*Longidorus nanus* Romanensko, 1993 – Russia, apple tree (*Malus* *domestica* Borkh.)

*Longidorus nevesi* Macara, 1985 – Portugal, *Juniperus phoenicea* L., *Pinus* *halepensis* Mill., *Cistis* *monspeliensis* L., *Pulcaria* *odora* (L.) Rchb., *Quercus* *coccifera* L., *Erica* *scoparia* L.

*Longidorus nirulai* Siddiqi, 1965 – India, *Solanum tuberosum* L.

*Longidorus* *oleae* Gutiérrez- Gutiérrez, Cantalapiedra-Navarrete, Monte-Borrego, Palomares-Rius & Castillo, 2013 – Spain, *Olea europaea* L.

*Longidorus olegi* Kankina & Metliskaya, 1983 – Russia, *Rubus idaeus* L.

*Longidorus orientalis* Loof, 1982 – Saudi Arabia, Iraq, date palm, grapevine

*Longidorus orongorngensis* Yeates, Van Etteger et Hooper, 1992 – New Zealand, conifer/broadleaf forest on a remnant river

*Longidorus paraelongatus* Altherr, 1974 – Germany, interstitial waters

*Longidorus paralaskaensis* Robbins & Brown, 1996 – USA, Alaska, rose

*Longidorus paralongicaudatus* Ye & Robbins, 2003 – USA, Arkansas, *Ulmus americana* L., *Acer* sp., *Quercus* sp*.*

*Longidorus paramirus* Darekar & Khan, 1982 – India, *Piper betel* L.

*Longidorus paramonile* Jacobs & Heyns, 1982 – S Africa, sugar cane

*Longidorus paravineacola* Ye & Robbins, 2003 – USA, Arkansas, *Acer* *negundo* L., *Ulmus* *americana* L., *Vitis* sp., *Maclura* *pomifera* (Raf. Schneid.), *Acer* sp., *Cercis* *canadensis* L., *Platanus* *occidentalis* L.

*Longidorus pauli* Lamberti, Molinari, De Luca, Agostinelli & Di Vito, 1999 – Syria, *Ficus carica* L.

*Longidorus pawneensis* Luc & Coomans, 1988 – USA, Colorado, grassland

*Longidorus piceicola* Liskova, Robbins & Brown, 1997 – Slovakia, *Picea abies* L.

*Longidorus picenus* Roca, Lamberti & Agostinelli, 1984 – Italy – *Mallus communis* L.

*Longidorus pini* Fe Andres & Arias, 1987 – Spain, *P. sylvestris*, *Quercus pyrenaica* L., *Juncus* sp.

*Longidorus pisi* Edward, Misra & Singh, 1964 – India, *Pisum sativum* L.

*Longidorus pius* Barsi & Lamberti, 2001 – Macedonia, *Carpinus orientalis* Mill.

*Longidorus poessneckensis* Altherr, 1974 – Germany, spring

*Longidorus profundorum* Hooper, 1966 – England, pears

*Longidorus protae* Roca & Bleve-Zacheo, 1977 – Italy, *Vitis* sp.

*Longidorus proximus* Sturhan & Argo, 1983 – Germany, arable soil

*Longidorus pseudoelongatus* Altherr, 1976 – Germany, interstitial water

*Longidorus psidii* Khan & Khan, 1972 – India, *Psidium guajava* L.

*Longidorus raskii* Lamberti & Agostinelli, 1993 – Switzerland *Mallus* *sylvestris* Mill

*Longidorus reisi* Roca & Bravo, 1993 – Portugal, *P. persica*

*Longidorus reneyii* Raina, 1966 – Kashmir, *Z. mays*

*Longidorus rotundicaudatus* Jacobs & Heyns, 1987 – South Africa, *V. vinifera*

*Longidorus rubi* Tomilin & Romanenko in Romanenko, 1993 *–* Ukraine, Poltava, *R.* *idaeus,* *M.* *domestica*

*Longidorus saginus* Khan, Seshardi, Weischer & Matten, 1971 – India, *Cocos nucifera* L.

*Longidorus seinhorsti* Peneva, Loof & Brown, 1998 – The Netherlands, river bank, grass

*Longidorus silvae* Roca, 1993– Italy, natural woodland

*Longidorus socialis* Singh & Khan, 1997 – India, *Pyrus communis* L.

*Longidorus spiralis* (Khan, Saha & Seshadri, 1972) Decraemer & Coomans, 2007 – India, *P. longifolia*

*Longidorus sturhani* Rubtsova, Subbotin, Brown & Moens, 2001 – Belgium, apple tree

*Longidorus sylphus* Thorne, 1939 – USA, Utah, forest soil

*Longidorus tabrizicus* Niknam, Pedram, Ghahremani Nejad, Ye, Robbins & Tanha Maafi, 2010 – Iran, *Rosa* sp.

*Longidorus taniwa* Clark, 1963 – New Zealand, *Weinmannia* *racemosa* L, *Holcus* *lanatus* L., *Agrostis* *tenuis* Sibth., pasture with *Juncus* sp., *Ranunculus* *acer* L. and *Lotus* *uliginosus* Schkuhr., *Carpodelus* *serratus* Forst.

*Longidorus tardicauda* Merzheevskaja, 1951 – Russia, *Linum* sp, *Solanum* sp.

*Longidorus tarjani* Siddiqi, 1962 – USA, Florida, *Quercus virginiana* Mill.

*Longidorus trapezoides* Nazira & Maqbool, 1995 – Pakistan, *S. tuberosum*

*Longidorus unedoi* Arias, Fe Andres & Navas, 1986 – Portugal, *Quercus faginea* L.

*Longidorus uroshis* Krnjaić, Lamberti, Krnjaić, Agostinelli & Radicci, 2000 – Montenegro, *Juniperus* *oxycedrus* L., *S. aspera*, *Hedera helix* L., *Myrthus communis* L.

*Longidorus vineacola* Sturhan & Weischer, 1954 – Germany, *V. riparia* Michx.

*Longidorus vinearum* Bravo & Roca, 1995 – Portugal, *V. vinifera*

*Longidorus waikouaitii* Yeates, Boag & Brown, 1997 – New Zealand, native forest remnants
