# Supplementary material for: Description of Longidorus cholevae sp. n. (Nematoda, Dorylaimida) from a riparian habitat in the Rila Mountains, Bulgaria
Source: Zookeys. 2013 Sep 9;(330):1–26. doi: 10.3897/zookeys.330.5750 (PMC3800803; doi:10.3897/zookeys.330.5750)
Supplement: Supplementary file 3 — A partial polytomous key to the species of Longidorus (doi: 10.3897/zookeys.330.5750.app2) File format: Microsoft Word Document (doc). [file ZooKeys-330-001-s002.doc]

A partial polytomous key to the species of *Longidorus* with long odontostyle (A45) and short tail (H1) based on the key by Chen et al. (1997) incorporating species described after 1997 and those transferred from other genera, see Appendix 1

|  | A | B | C | D | E | F | G | H | I | **J** | **K** |
| --- | --- | --- | --- | --- | --- | --- | --- | --- | --- | --- | --- |
|  |  |  |  |  |  |  |  |  |  |  |  |
| *L. igoris* | 24 | 1 | 23 | 1 | 3 | 23 | 23 | 1 | 1 | **1** | **3** |
| *L. athesinus* | 3 | 23 | 3 | 1 | 3 | 23 | 12 | 12 | 2 | **1** | **3** |
| *L. vineacola* | 3 | 3 | 23 | 2 | 3 | 4 | 3 | 1 | 2 | **1** | **6(7)** |
| *L. glycines* | 3 | 4 | 2 | 3 | 2 | 34 | 24 | 12 | 2 | **1** | **4** |
| *L. balticus* | 34 | 12 | 2 | 3 | 3 | 34 | 23 | 1 | 2 | **1** | **?** |
| *L. orientalis* | 34 | 1 | 23 | 1 | 2 | 23 | 2 | 1 | 12 | **1** | **3** |
| *L. milanis* | 34 | 2 | 3 | 3 | 3 | 2 | 1 | 2 | 2 | **1** | **7** |
| *L henanus* | 34 | 23 | 34 | 2 | 3 | 3 | 23 | 12 | 1 | **1** | **6** |
| *L. crataegi* | 34 | 234 | 3 | 1 | 3 | 34 | 12 | 1 | 2 | **1** | **7** |
| *L. jagerae* | 34 | 12 | 5 | 4 | 1 | 2 | 2 | 12 | 1 | **?** | **?** |
| *L. raskii* | 34 | 3 | 3 | 1 | 2 | 34 | 12 | 1 | 2 | **1** | **23** |
| *L. goodeyi* | 34 | 3 | 3 | 2 | 3 | 34 | 12 | 1 | 1 | **1** | **7** |
| *L. caespiticola* | 34 | 3 | 34 | 1 | 4 | 34 | 12 | 1 | 2 | **1** | **3** |
| *L. taniwa* | 34 | 3 | 45 | 1 | 2 | 2 | 12 | 1 | 2 | **2** | **12** |
| *L. paravineacola* | 34 | 45 | 23 | 3 | 2 | 35 | 24 | 1 | 1 | **?** | **?** |
| *L. moesicus* | 35 | 12 | 23 | 1 | 3 | 34 | 23 | 12 | 1 | **1** | **6** |
| *L. apulus* | 35 | 23 | 23 | 3 | 23 | 34 | 3 | 2 | 1 | **1** | **6** |
| *L. doonensis* | 35 | 1 | 5 | 1 | 2 | 2 | 1 | 1 | 1 | **?** | **?** |
| *L. pauli* | 4 | 23 | 23 | 3 | 23 | 4 | 3 | 1 | 2 | **1** | **7** |
| *L. tardicauda* | 4 | 1 | ? | 3 | 1 | 23 | 2 | 1 | 1 | **?** | **?** |
| *L. iranicus* | 4 | 12 | 3 | 1 | 2 | 3 | 2 | 1 | 1 | **1** | **6** |
| *L. olegi* | 4 | 2 | 3 | 1 | 2 | 4 | 2 | 1 | 2 | **?** | **?** |
| *L. fasciatus* | 4 | 2 | 3 | 1 | 3 | 34 | 23 | 1 | 1 | **?** | **3** |
| *L. trapezoides* | 4 | 2 | 3 | 2 | 3 | 34 | 23 | 1 | 1 | **?** | **?** |
| *L. jonesi* | 4 | 2 | 5 | 1 | 1 | 2 | 1 | 1 | 1 | **2** | **?** |
| *L. boshi* | 4 | 2 | 45 | 2 | 1 | 2 | 12 | 1 | 12 | **?** | **?** |
| *L. arthensis* | 4 | 23 | 3 | 1 | 2 | 3 | 12 | 12 | 2 | **1** | **67** |
| *L. crassus* | 4 | 3 | 3 | 3 | ? | 24 | 2 | 1 | 1 | **1** | **23?** |
| *L. diadecturus* | 4 | 23 | 5 | 2 | 5 | 2 | 12 | 1 | 1 | **1** | **6** |
| *L. proximus* | 4 | 3 | 3 | 3 | 1 | 34 | 23 | 1 | 12 | **1** | **6** |
| *L. magnus* | 4 | 34 | 4 | 1 | 3 | 5 | 12 | 1 | 1 | **1** | **5** |
| *L. kuiperi* | 4 | 5 | 23 | 3 | 1 | 34 | 34 | 1 | 2 | **1** | **1** |
| *L. heynsi* | 4 | 4 | 5 | 2 | 2 | 4 | 2 | 1 | 2 | **?** | **?** |
| *L. hangzhouensis* | 45 | 1 | (3)4 | 1 | 4 | 2 | 1 | 1 | 1 | **1** | **?** |
| *L. pseudoelongatus* | 45 | 2 | 23 | 4 | 4 | 3 | 12 | 1 | 1 | **?** | **?** |
| *L. baeticus* | 45 | 2 | 3 | 1 | 3 | 4(35) | 12 | 1 | 2 | **1** | **23** |
| *L. himalayaensis* | 45 | 2 | 5 | 2 | 2 | 2 | 2 | 1 | 1 | **?** | **?** |
| *L. monegrensis* | 45 | 23 | 23 | 2 | 5 | 45 | 23 | 1 | 2 | ? | 6 |
| *L. apuloides* | 45 | 23 | 3 | 2 | 3 | 45 | 34 | 12 | 2 | **1** | **6** |
| *L. silvae* | 45 | 23 | 34 | 1 | 3 | 34 | 2(3) | 1 | 1 | **1** | **7** |
| *L. iuglandis* | 45 | 23 | 34 | 12 | 2 | 34 | 12 | 1 | 2 | **1?** | **2** |
| *L. lignosus* | 45 | 3 | 3 | 3 | 1 | 23 | 1 | 1 | 2 | **?** | **?** |
| *L. macromucronatus* | 45 | 3 | 5 | 3 | 1 | 2 | 2 | 1 | 1 | **1** | **56** |
| *L. oleae* | 45 | 3(4) | 3(3) | 3 | 3 | 4(35) | 2 (1) | 1 | 2 | **1** | **3** |
| *L. vinearum* | 45 | 35 | 34 | 2 | 3 | 45 | 12 | 1 | 2 | **1** | **6** |
| ***L. cholevae*** | **45** | **4** | **23** | **1** | **5** | **34** | **12** | **1** | **2** | **1** | **56** |
| *L. kheirii* | 45 | 345 | 34 | 1(7) | 2 | 34 | 12 | 1 | 2 | **1** | **7** |
| *L. macrosoma* | 46 | 4 | 34 | 3 | 4 | 45 | 13 | 1 | 2 | **1** | **7** |
| *L. cylindricaudatus* | 5 | 2 | 3 | 2 | 2 | 23 | 12 | 1 | 1 | **1?** | **6** |
| *L. paraelongatus* | 5 | 23 | 3 | 23 | 1 | 34 | 2 | 2 | 2 | **?** | **?** |
| *L. israelensis* | 5 | 3 | 3 | 3 | 1 | 4 | 23 | 1 | 1 | **?** | **?** |
| *L. macroteromucronatus* | 5 | 3 | 4 | 1 | ? | 4 | 12 | 1 | 1 | **?** | **?** |
| *L. poessneckensis* | 5 | 34 | 3 | 1 | 4 | 35 | 2 | 1 | 12 | **1** | **3** |
| *L. major* | 5 | 45 | 34 | 2 | 3 | 45 | 2 | 1 | 1 | **1** | **7** |
| *L. cedari* | 56 | 2 | 5 | 1 | 1 | 23 | 1 | 1 | 12 | ? | ? |
| *L. cretensis* | 56 | 34 | 4 | 3 | 1 | 34 | 34 | 1 | 12 | **1** | **6** |
| *L. nevesi* | 56 | 34 | 3(4) | 1 | 3 | 35 | 12 | 1 | 2 | **1** | **3** |
| *L. picenus* | 56 | 3(4) | 34 | 1 | 3 | 34 | 2 | 1 | 2 | **1** | **7** |
| *L. mindanaoensis* | 5(6) | 34 | 3(4) | 1 | 4 | 3(4) | (1)2 | 1 | 2 | **1** | **2** |
| *L. saginus* | 56 | 45 | 23 | 3 | 3 | 23 | 23 | 1 | 2 | **?** | **?** |
| *L. pius* | 56 | 45 | 34 | 1 | 1 | 23 | 1 | 1 | 1 | **1** | **7** |
| *L. helveticus* | 56 | 45 | 34 | 1 | 4 | 34 | 1 | 1 | 2 | **1** | **7** |
| *L. uroshis* | 56 | 24 | 34 | 3 | 23 | 34 | 2 | 1 | 2 | **1** | **7** |
| *L. carniolensis* | 56 | 4 | 4 | 3 | 4 | 345 | 1 | 1 | 2 | **1** | **2** |
| *L. litchii* | 57 | 2 | 5 | 2 | 2 | 23 | 1(2) | 1 | 2 | 1 | 7 |
| *L. americanus* | 57 | 5 | 34 | 3 | 2 | 34 | 23 | 12 | 12 | **1** | **6** |
